# Supplementary figures and images for: Chemical genetics strategy to profile kinase target engagement reveals role of FES in neutrophil phagocytosis
Source: Nat Commun. 2020 Jun 25;11:3216. doi: 10.1038/s41467-020-17027-5 (PMC7316778; doi:10.1038/s41467-020-17027-5)

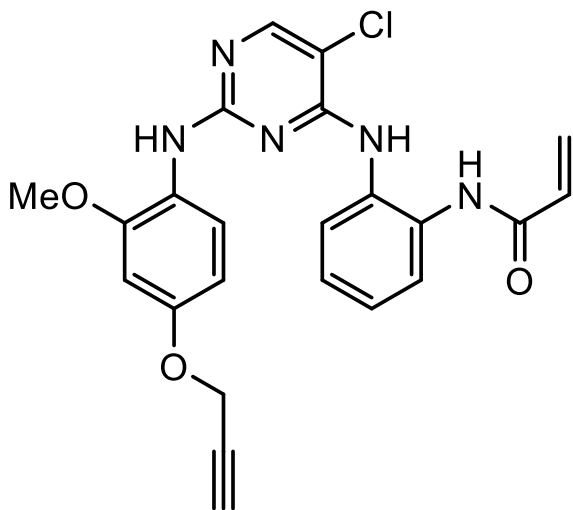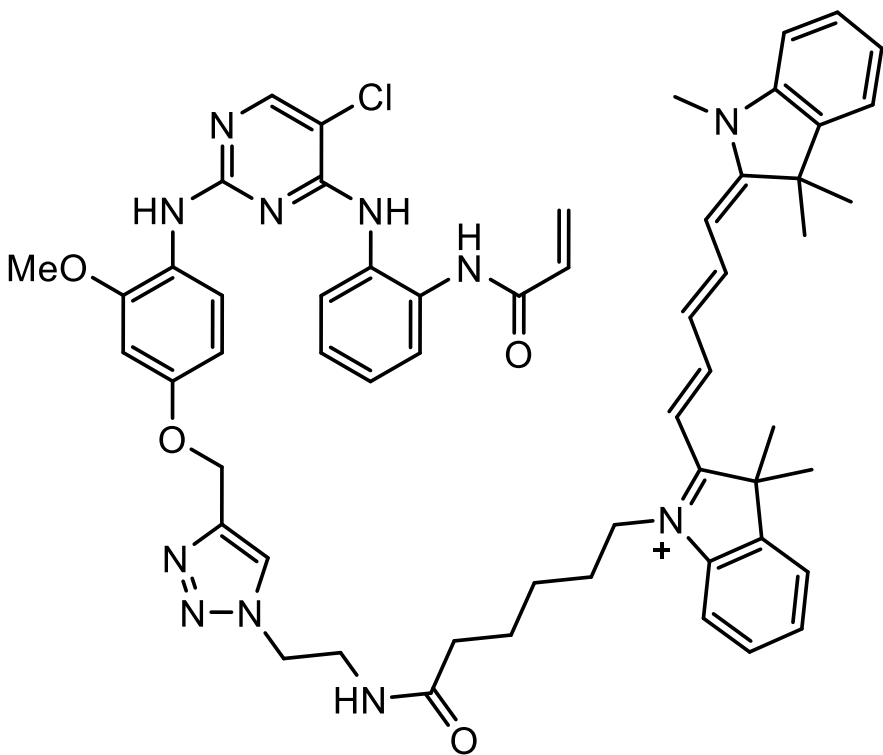

Supplement: Supplementary file 1 — ChemDraw File [file 41467_2020_17027_MOESM1_ESM.pdf]
