# Supplementary material for: Chemical genetics strategy to profile kinase target engagement reveals role of FES in neutrophil phagocytosis
Source: Nat Commun. 2020 Jun 25;11:3216. doi: 10.1038/s41467-020-17027-5 (PMC7316778; doi:10.1038/s41467-020-17027-5)
Supplement: Supplementary file 2 — Supplementary Information [file 41467_2020_17027_MOESM2_ESM.pdf]

Supplementary Information accompanying

Chemical genetics strategy to profile kinase target engagement reveals role of FES in neutrophil phagocytosis

T. van der Wel *et al.*

Supplementary Figures

Supplementary Tables

Supplementary Methods

Supplementary Notes

Supplementary Discussion

Supplementary References

Supplementary Figures

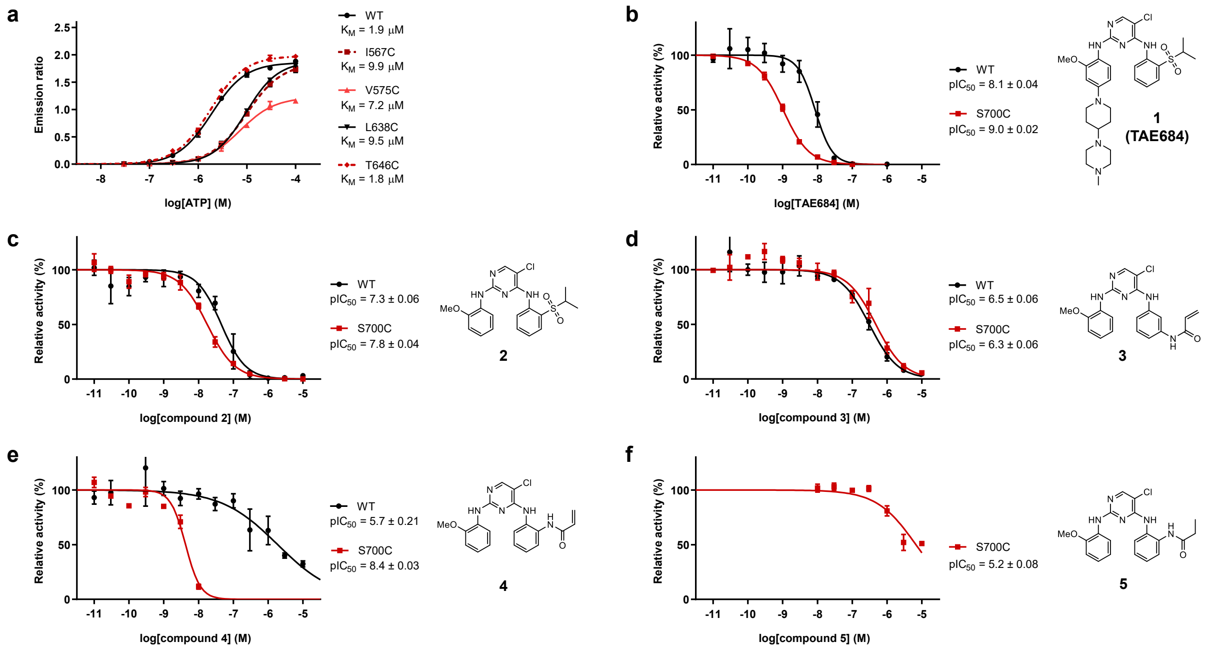

Supplementary Figure 1. Determination of ATP K_M for other active FES mutants and concentration-response curves of inhibitors against FES^{WT} and FES^{S700C}.

(**a**) Determination of ATP K_M for FES^{WT} and other FES mutants with >50% relative activity as determined in Fig. 2b.

(**b-f**) Concentration-response curves of inhibitors against FES^{WT} and FES^{S700C} as determined in TR-FRET assay with 5 μM ATP. Compound code and structure is depicted right of the corresponding curve. Data represent means ± SEM (n = 3). Source data are provided as a Source Data file.

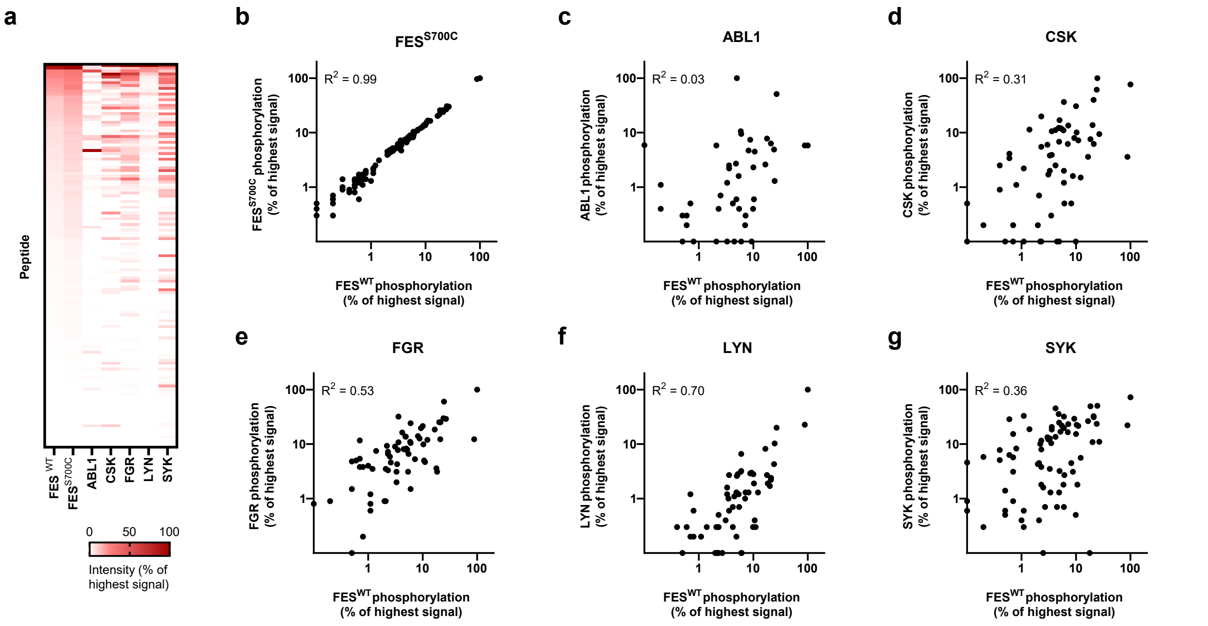

Supplementary Figure 2. Peptide substrate profile of FES is distinct from other non-receptor tyrosine kinase profiles.

(**a**) Heatmap representation of relative phosphorylation intensities for FES^{WT}, FES^{S700C} and five non-receptor tyrosine kinases. Scale shows relative signal intensity from minimum (0%, white) to maximum (100%, red).

(**b-g**) Correlations between the peptide substrate profiles of FES^{WT} and FES^{S700C} (**b**), ABL1 (**c**), CSK (**d**), FGR (**e**), LYN (**f**) and SYK (**g**) as determined using the PamChip® microarray. Peptides were filtered for those with ATP-dependent signal and normalized to the highest signal intensity per kinase (n = 3). Source data are provided as a Source Data file.

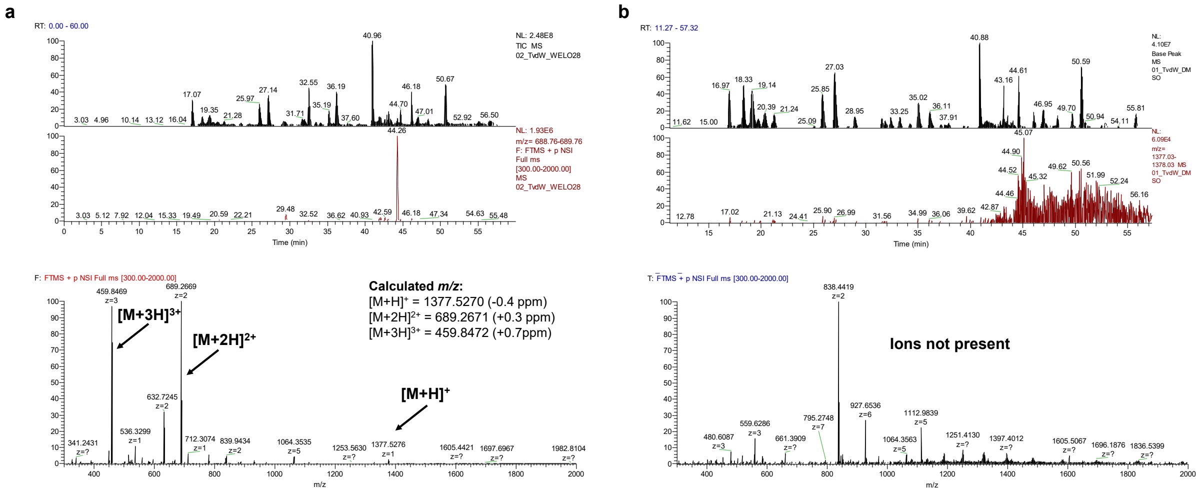

Supplementary Figure 3. LC-MS elution profiles of FES^{S700C} incubated with WEL028 (a) or vehicle (b). Expected precursor ions and corresponding calculated m/z values are indicated. Ions were not present in vehicle-treated control sample.

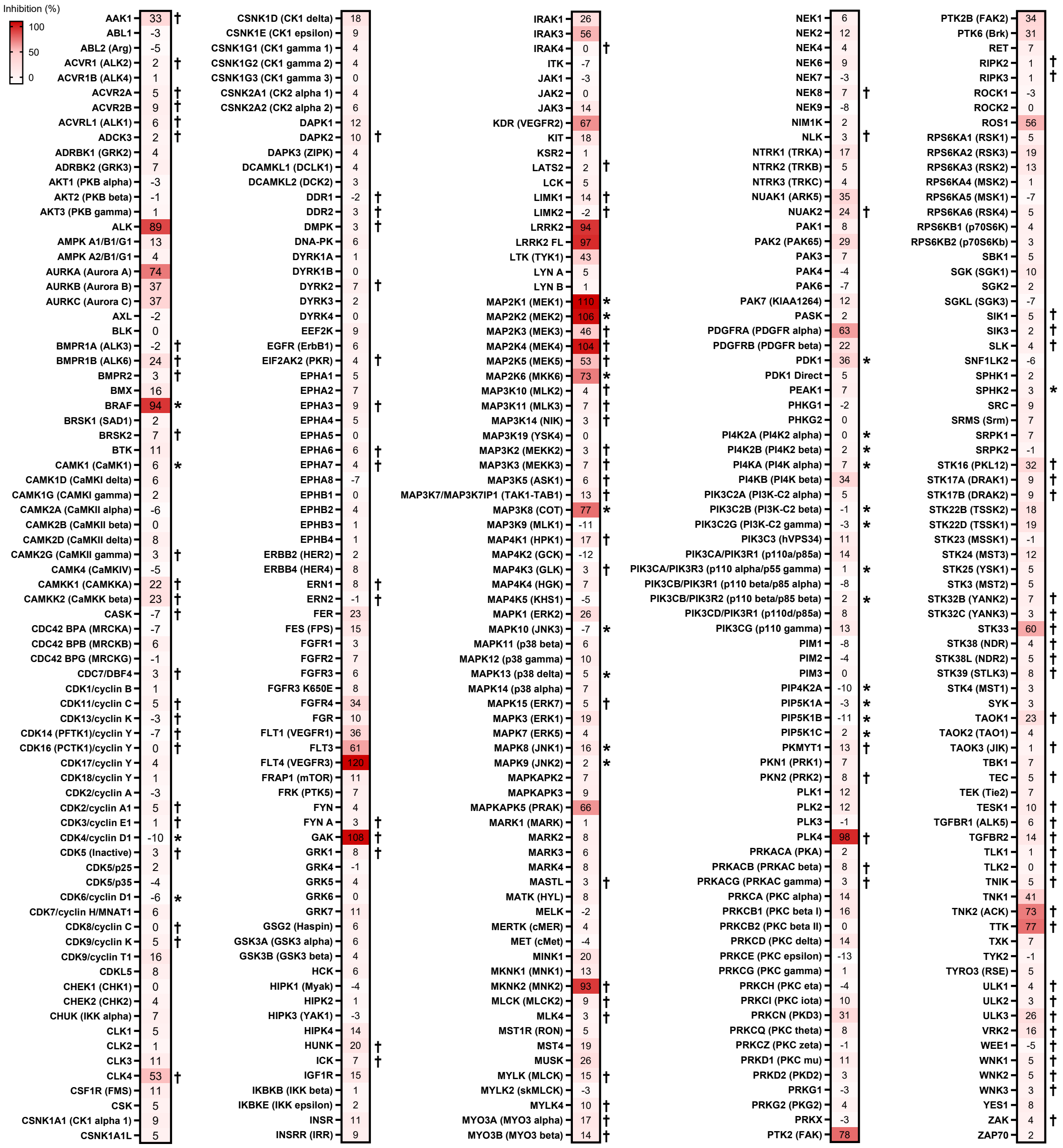

Supplementary Figure 4. Single-point selectivity screen of WEL028 on a panel of 380 kinases. All data were obtained from SelectScreen™ selectivity profiling service. Assays were performed at 1 μM WEL028 with 1 h preincubation. The ATP concentration was equal to the kinase K_M, except for those indicated († = LanthaScreen technology, no ATP; * = 100 μM ATP; ** = 10 μM ATP). Values represent mean percentage inhibition compared to vehicle-treated control (n = 2). Scale shows percentage inhibition from minimum (0%, white) to maximum (100%, red). Source data are provided as a Source Data file.

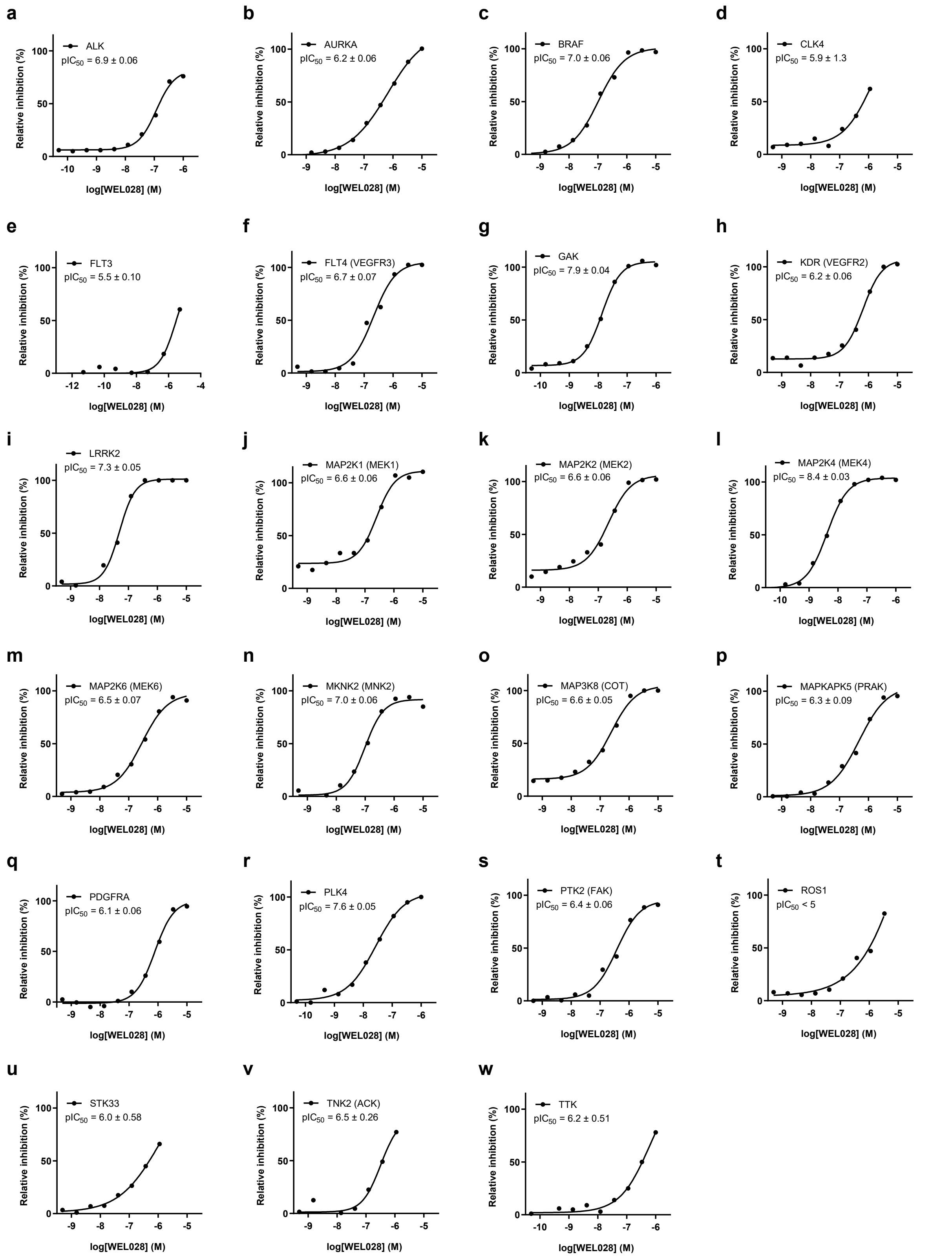

Supplementary Figure 5. Concentration-response curves of WEL028 against a representative selection of kinases with >50 % inhibition at 1 μM in initial single-dose screen. Data (means, n = 2) were obtained from SelectScreen™ selectivity profiling service. Assays were performed with 1 h preincubation and concentration of ATP was selected to be equal to the K_M, unless indicated otherwise in Supplementary Figure 4. Source data are provided as a Source Data file.

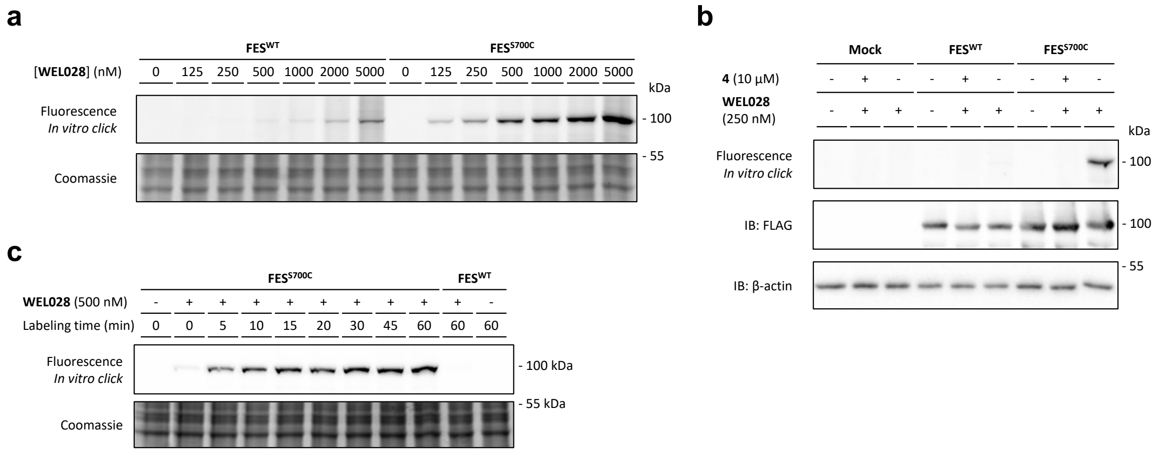

Supplementary Figure 6. Two-step labeling of FES^{S700C} by WEL028 conjugated to Cy5-azide *in vitro* using click chemistry.

(**a**) Dose-dependent labeling of recombinantly expressed full-length FES^{S700C} but not FES^{WT} in HEK293T cell lysate. Lysates were incubated with WEL028 (indicated concentration, 30 min, rt), followed by addition of click mix containing Cy5-azide (2 eq., 30 min, rt). Samples were resolved by SDS-PAGE, followed by in-gel fluorescence scanning.

(**b**) Two-step labeling by WEL028 is specific and exclusive for FES^{S700C}. Recombinantly expressed FES in HEK293T cell lysate was preincubated with vehicle or compound **4** (10 µM, 30 min, rt), followed by incubation with two-step probe WEL028 (250 nM, 30 min, rt) and click mix containing Cy5-azide (2 eq., 30 min, rt). Samples were resolved by SDS-PAGE, followed by in-gel fluorescence scanning. Protein expression was verified by immunoblot against a C-terminal FLAG-tag and β-actin as loading control.

(**c**) WEL028 labeling kinetics for FES^{S700C}. Lysates were incubated with WEL028 (500 nM, indicated time, rt) and processed as in **a**. Complete labeling was achieved after 15 min and this labeling was stable up to 60 min. Source data are provided as a Source Data file.

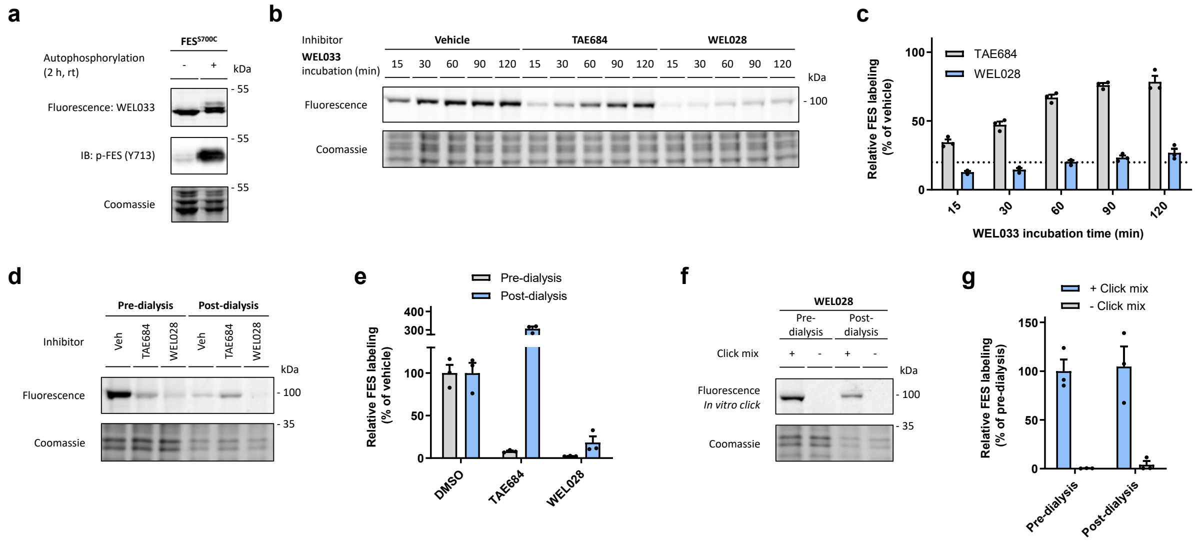

Supplementary Figure 7. Characterization of irreversible, covalent binding mode of WEL028 by probe displacement and inhibitor washout experiments.

(**a**) WEL033 labels FES^{S700C} regardless of its autophosphorylation status. *E. coli* BL21(DE3) recombinantly co-expressing truncated FESS700C (SH2-KD) and YopH were lysed, after which part of the lysate was subjected to autophosphorylation (2 h, rt). Lysates were labeled with WEL033 (250 nM, 30 min, rt) and analyzed by in-gel fluorescence and immunoblot using anti-phospho-FES Y713 antibody.

(**b-c**) WEL033 outcompetes TAE684 but not WEL028 binding over time. Lysate was incubated with vehicle, TAE684 or WEL028 at the respective IC_{80}-concentration (20% remaining activity; TAE684: 82 nM, WEL028: 27 nM; 30 min, rt), followed by incubation with WEL033 (1 µM, indicated time, rt). Band intensities were normalized to vehicle-treated control at same time point (n = 3).

(**d-e**) Sustained FES^{S700C} inhibition by WEL028 but not TAE684 after overnight dialysis. Lysates were treated with vehicle, TAE684 or WEL028 as in panel **b** and pre-dialysis samples were directly flash-frozen after incubation. Residual lysate was dialyzed overnight at 4°C. Pre- and post-dialysis samples were then treated with WEL033 (250 nM, 30 min, rt). Band intensities were normalized to vehicle-treated control (n = 3).

(**f-g**) Two-step labeling of WEL028-bound FES^{S700C} before and after dialysis. Samples were processed as in panel **d**, but conjugated to BODIPY-azide using click chemistry (2 eq., 30 min, rt). Band intensities were normalized to pre-dialysis control (n = 3). Data represent means ± SEM. Source data are provided as a Source Data file.

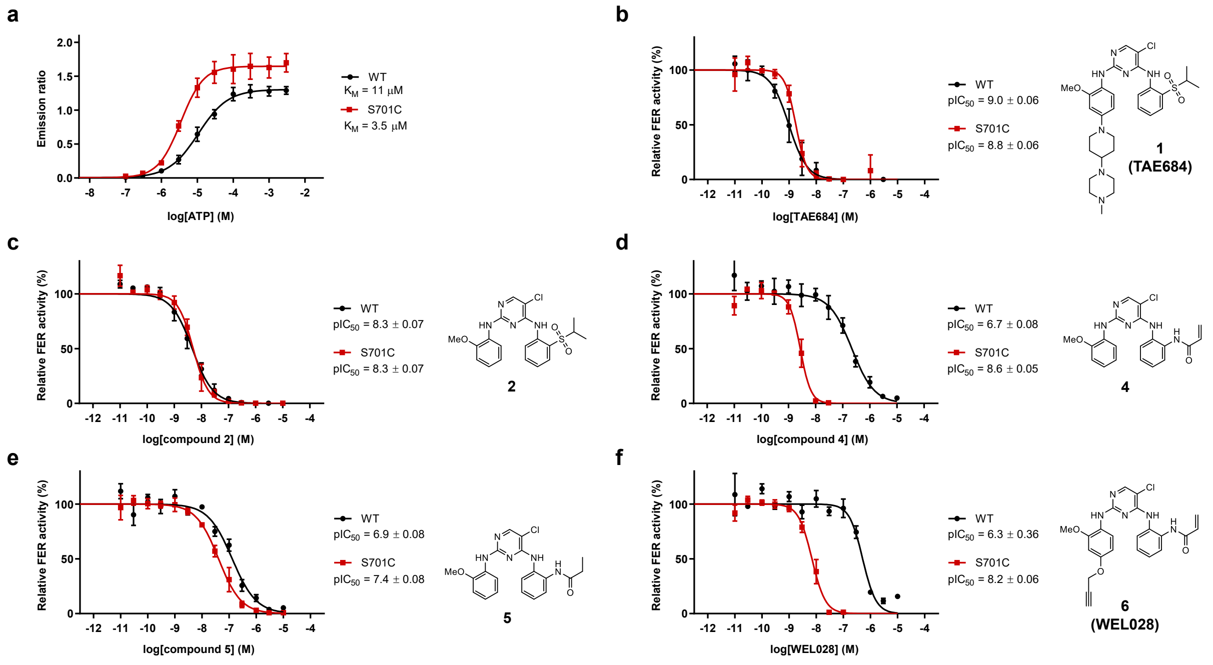

Supplementary Figure 8. Determination of ATP K_M for FER^{WT} and FER^{S701C} and concentration-response curves of inhibitors against FER^{WT} and FER^{S701C}.

(**a**) Determination of ATP K_M for FER^{WT} and FER^{S701C}.

(**b-f**) Concentration-response curves of inhibitors against FER^{WT} and FER^{S701C} as determined in TR-FRET assay. Final ATP concentration was 12 μM and 1 μM for FER^{WT} and FER^{S701C}, respectively. Compound code and structure is depicted right of the corresponding curve. Data represents means ± SEM (n = 3). Source data are provided as a Source Data file.

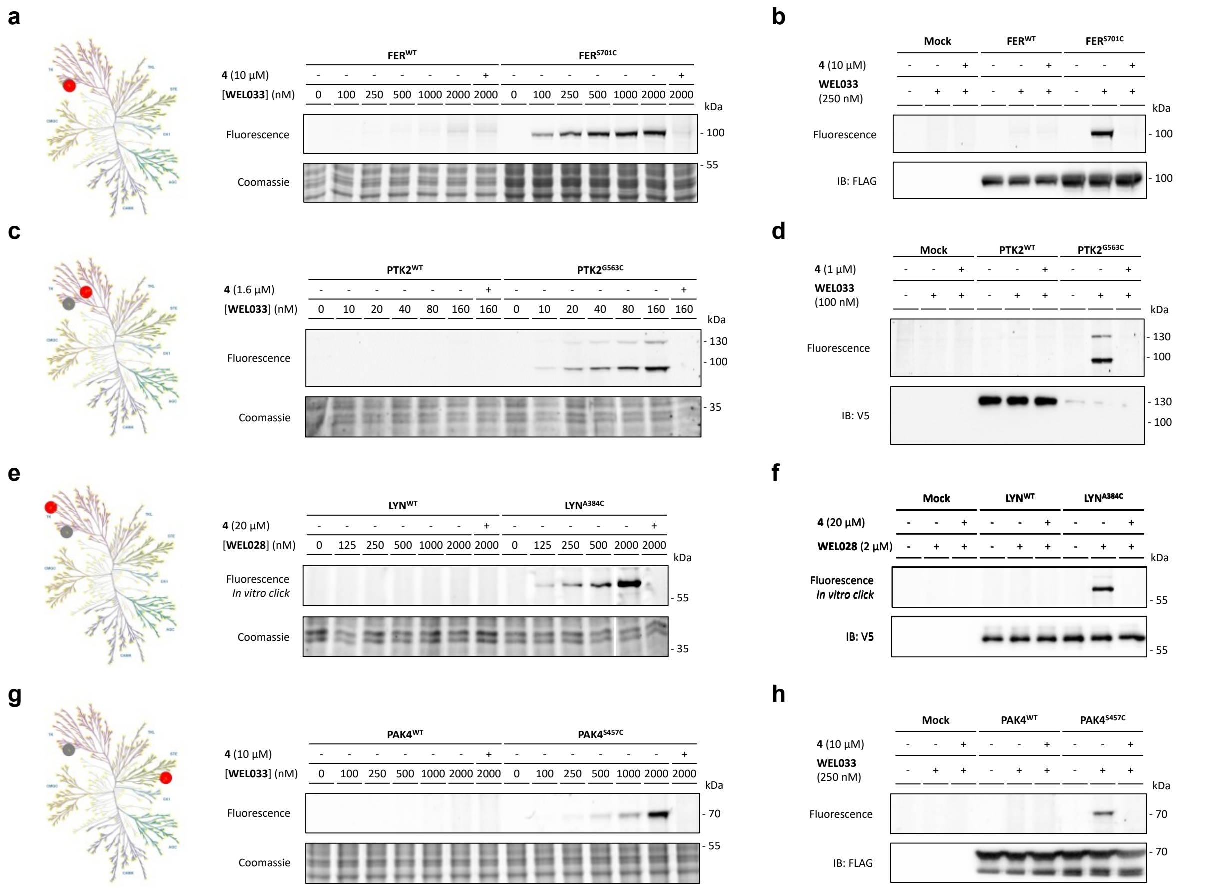

Supplementary Figure 9. Applicability of chemical genetic strategy on various kinases harboring DFG-1 residues mutated into cysteines.

(**a, c, e, g**) Dose-dependent and specific labeling of DFG-1 cysteine mutants but not wild-type kinases by complementary probes. Recombinantly expressed kinase (**a**: FER, **c**: PTK2, **e**: LYN, **g**: PAK4) in HEK293T cell lysate were preincubated with vehicle or **4** (indicated concentration, 30 min, rt), followed by incubation with probe (**a**, **c**, **g**: one-step probe WEL033, **e**: two-step probe WEL028; indicated concentrations, 30 min, rt). For **e**, samples were then conjugated to Cy5-azide using click chemistry. Homology-based similarity of corresponding kinase (red) to FES (gray) is visualized in kinome tree illustrations.

(**b, d, f, h**) Labeling by complementary probes is specific and exclusive for DFG-1 cysteine mutants. Samples were treated as aforementioned, but at the optimal probe concentration (indicated, 30 min, rt). Protein expression was verified by immunoblot against a C-terminal FLAG-tag or V5-tag. Of note, PTK2 migrated a two bands of which only the upper band was detected by immunoblot. Kinome illustrations were rendered using KinMap (www.kinhub.org/kinmap), reproduced courtesy of Cell Signaling Technology, Inc. www.cellsignal.com). Source data are provided as a Source Data file.

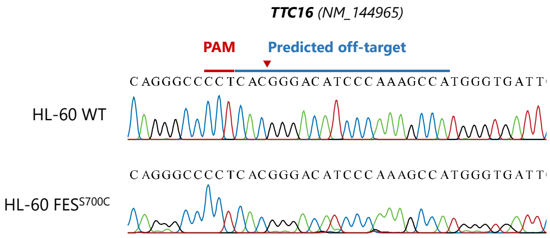

Supplementary Figure 10. Analysis of putative sgRNA off-target site. Analysis of the only predicted coding off-target of the sgRNA target sequence employed for FES^{S700C} mutagenesis, located in the *TTC16* gene. Genomic region surrounding putative off-target site was amplified by PCR, followed by Sanger sequencing analysis. No off-target gene editing events were observed.

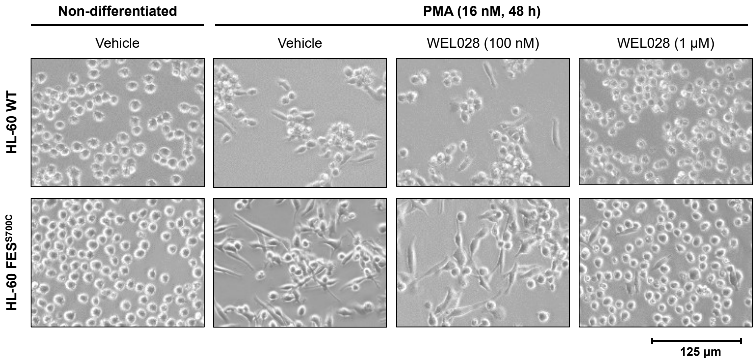

Supplementary Figure 11. Morphological inspection of WT and FES^{S700C} HL-60 cells after PMA-induced differentiation towards macrophages. Cells were treated with vehicle or indicated concentrations of WEL028 during PMA-induced differentiation toward macrophages. Wild-type and FES^{S700C} HL-60 cells display similar macrophage morphology. Cells display macrophage morphology at 100 nM WEL028, but morphology is similar to non-differentiated cells at 1 µM WEL028. Shown images are representative for multiple acquired images at 20x magnification from replicates (n = 3).

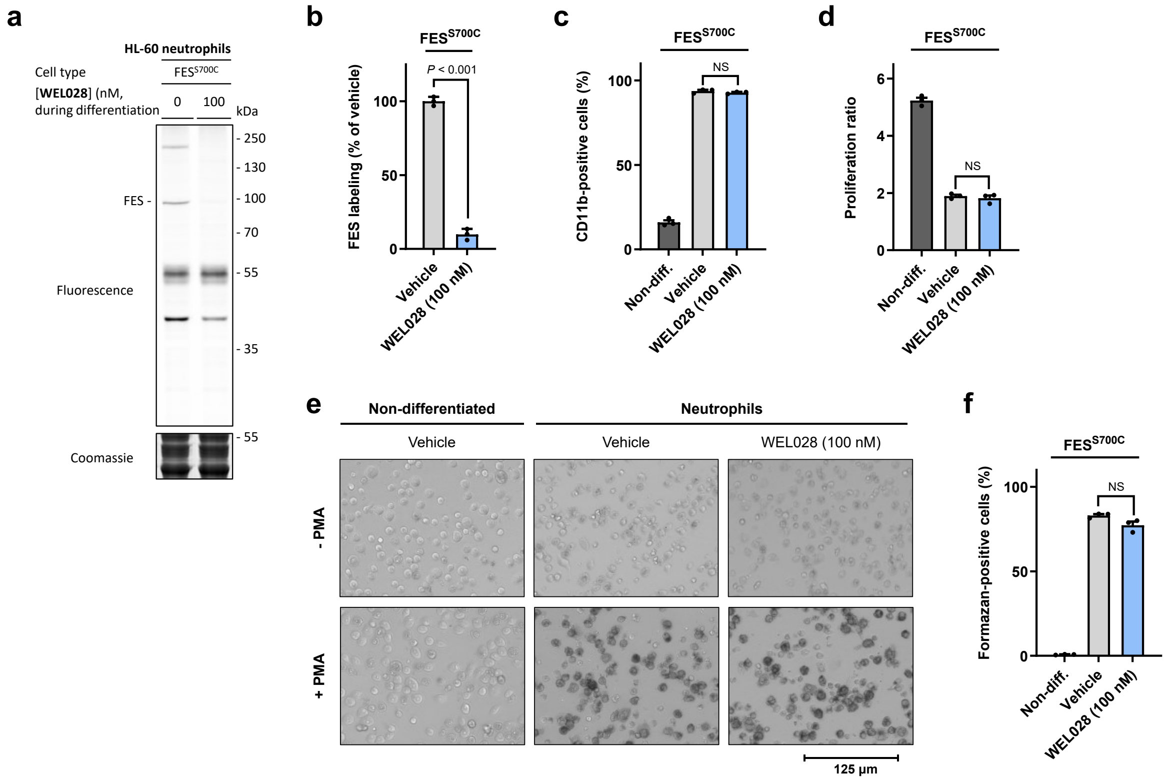

Supplementary Figure 12. FES activity is dispensable for differentiation of HL-60 cells into neutrophils.

(**a**-**b**) Target engagement profile of WEL028 on FES^{S700C} HL-60 cells treated during neutrophil differentiation. Cells were pretreated with vehicle or WEL028 (100 nM, 1 h) prior to induction of differentiation towards neutrophils with ATRA (1 µM) and DMSO (1.25%, 72 h). Medium was refreshed with growth medium containing WEL028 and ATRA/DMSO every 24 h to maintain full FES inhibition. Lysates were incubated with WEL033 (1 µM, 30 min, rt). Band intensities were normalized to vehicle-treated control (n = 3).

(**c**) CD11b surface expression analyzed by flow cytometry. Threshold for CD11b-positive cells was determined using isotype control antibody (n = 3).

(**d**) Proliferation of FES^{S700C} HL-60 cells subjected to neutrophil differentiation. Proliferation ratio: live cell number after differentiation divided by live cell number before differentiation (n = 3).

(**e**-**f**) Ability of HL-60 neutrophils to induce oxidative burst upon PMA stimulation. FES^{S700C} HL-60 cells were incubated with 0.1% nitroblue tetrazolium (NBT) with or without PMA (1.6 µM, 1 h, 37°C), imaged by phase contrast microscopy (20x magnification) and cells positive for formazan deposits were counted (n = 3, with 3 different counted fields per replicate). Data represent means ± SEM. Statistical analysis: two-tailed *t*-test: *** $P < 0.001$, NS if $P > 0.05$. Source data are provided as a Source Data file.

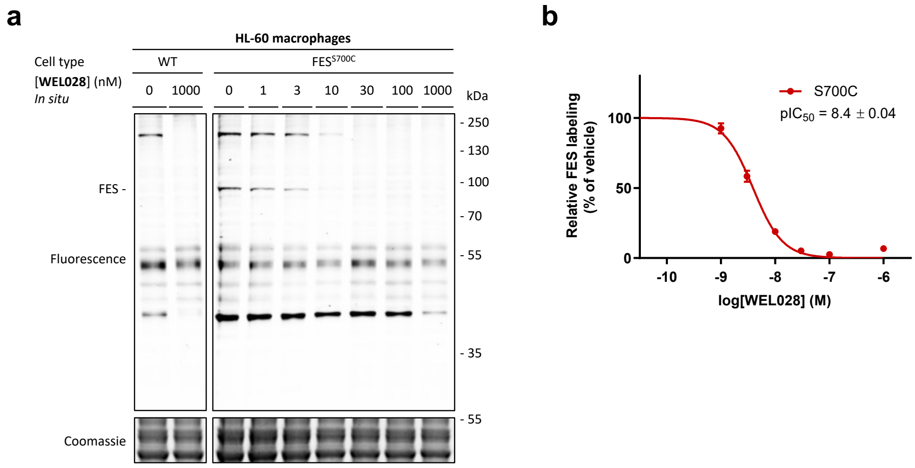

Supplementary Figure 13. WEL028 dose-dependently engages endogenous FES *in situ*.
(**a-b**) Live FES^{S700C} HL-60 macrophages were treated with various concentrations of WEL028 for 1 h and harvested. Lysates were incubated with WEL033 (1 μM, 30 min, rt). Band intensities were normalized to vehicle-treated control (n = 3). Data represent means ± SEM. Source data are provided as a Source Data file.

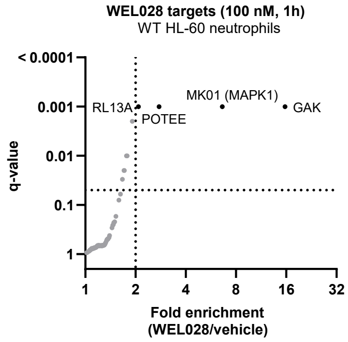

Supplementary Figure 14. Chemical proteomics-based identification of WEL028 kinase targets at 100 nM in WT HL-60 neutrophils. Kinases with > 2-fold enrichment compared to vehicle control (q < 0.05) were designated as targets (shown in black). Values represent means of fold enrichment (n = 3). Source data are provided as a Source Data file.

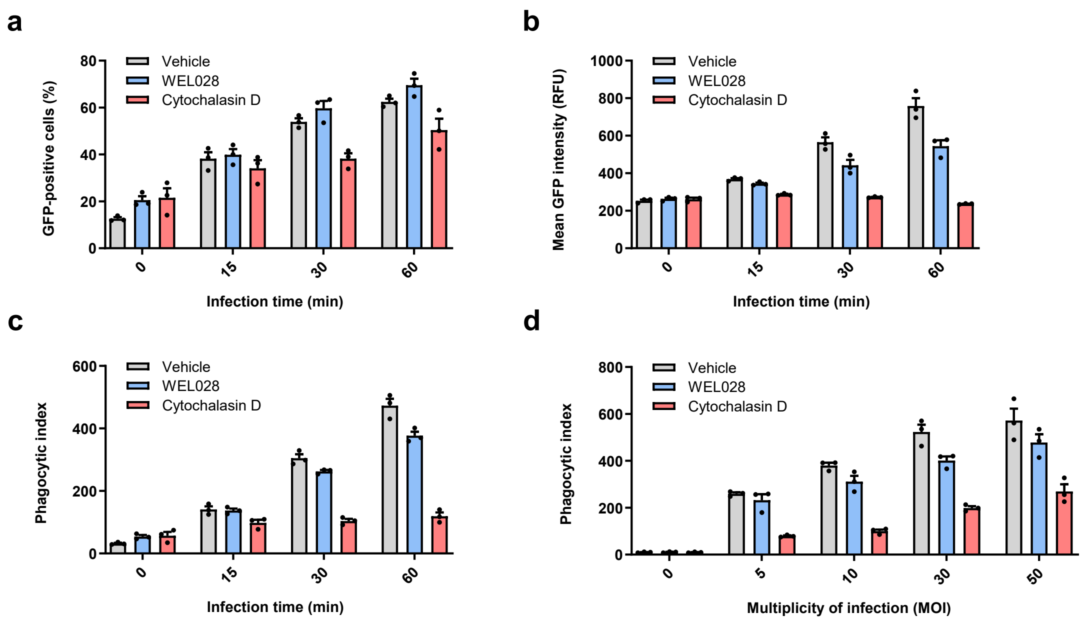

Supplementary Figure 15. Phagocytic uptake of *E. coli* by HL-60 FES^{S700C} neutrophils at variable multiplicity of infection (MOI) and infection time.

(**a-c**) HL-60 FES^{S700C} neutrophils were incubated with vehicle, WEL028 (100 nM) or Cytochalasin D (10 µM) for 1 h, after which GFP-expressing E. coli were added at MOI of 30. After indicated times, cells were washed and fixed (1% PFA, 15 min, 4 °C), followed by flow cytometry analysis (n = 3). Phagocytic index (**c**) was calculated as fraction of GFP-positive cells (number of phagocytic cells, **a**) multiplied by GFP MFI (number of phagocytized bacteria, **b**).

(**d**) Neutrophils were treated as in panel **a-c**, but with a variable MOI for 1 h (n = 3). Data represent means ± SEM. Source data are provided as a Source Data file.

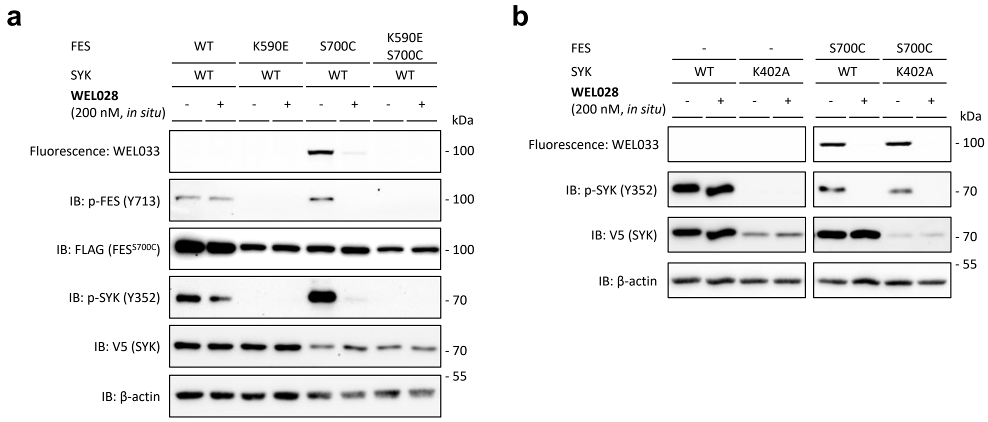

Supplementary Figure 16. SYK Y352 phosphorylation is exclusively mediated via FES in co-transfected U2OS cells, but occurs via SYK autophosphorylation in absence of FES.

(**a**) Co-transfection of SYK with kinase-dead FES^{K590E} variants abolishes SYK Y352 phosphorylation. U2OS cells were co-transfected with SYK and FES^{WT}, FES^{S700C} or the corresponding kinase-dead K590E variants. After 48 h, cells were incubated with vehicle or WEL028 (200 nM, 1 h) and lysed. Lysates were incubated with WEL033 (250 nM, 30 min, rt) and analyzed by in-gel fluorescence and immunoblot (n = 3).

(**b**) SYK autophosphorylates at Y352 in absence of co-transfected FES. U2OS cells were transfected with only SYK (WT or kinase-dead variant K402A that is incapable of undergoing autophosphorylation) or co-transfected with SYK^{WT}/SYK^{K402A} and FES^{S700C}. After 48 h, cells were incubated with vehicle or WEL028 (200 nM, 1 h) and lysed. Lysates were incubated with WEL033 (250 nM, 30 min, rt) and analyzed by in-gel fluorescence and immunoblot (n = 3). Source data are provided as a Source Data file.

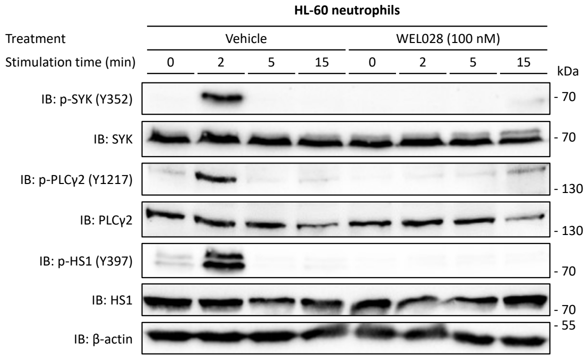

Supplementary Figure 17. FES rapidly phosphorylates endogenous SYK Y352 and downstream substrates HS1 Y397 and PLCγ2 Y1217 in HL-60 neutrophils infected with *E. coli*. HL-60 neutrophils were incubated with vehicle or WEL028 (100 nM, 1 h), followed by addition of GFP-expressing *E. coli* B834 (MOI = 30, 0-2-5-15 min, 37°C) and immunoblot analysis (n = 3). Source data are provided as a Source Data file.

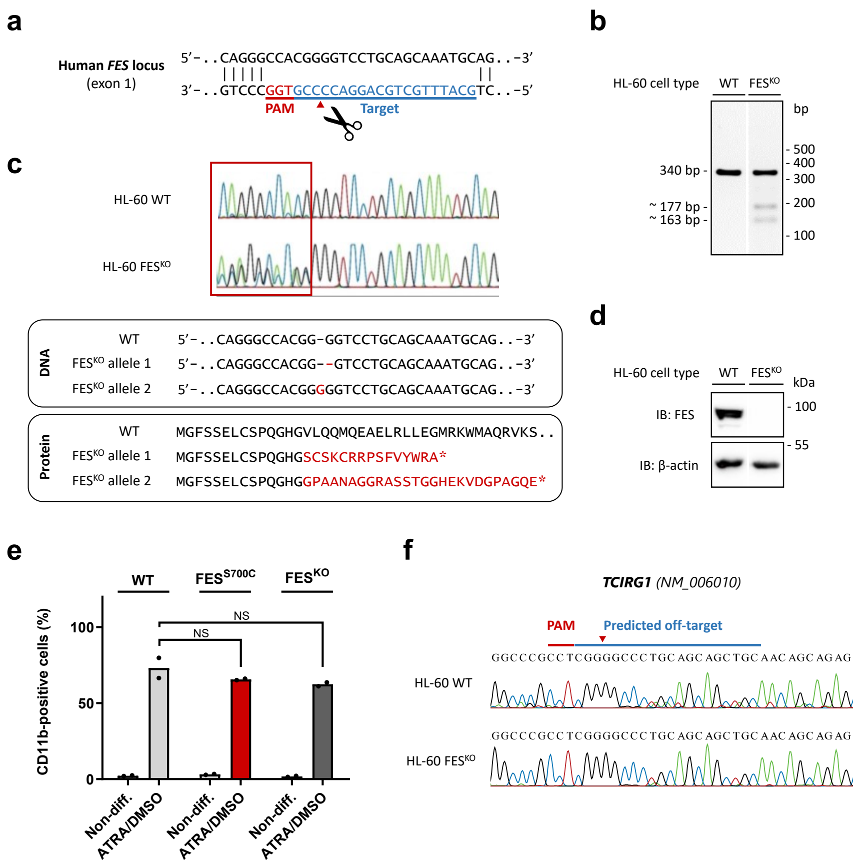

Supplementary Figure 18. Generation and validation of a FES knockout HL-60 cell line using CRISPR/Cas9.

(**a**) CRISPR/Cas9 gene editing strategy for FES knockout in HL-60 cells. Selected sgRNA (bold blue) directs Cas9 to cleave at predicted site (red triangle). Repair of the induced double-strand break by non-homologous end-joining (NHEJ) results in deletions or insertions that can lead to a translational frameshift and introduction of a premature stop codon. PAM: protospacer-adjacent motif.

(**b**) T7 endonuclease I (T7E1) assay for identification of HL-60 FES^{KO} clone. Genomic region was amplified by PCR and amplicons were analyzed using T7E1 assay. Expected fragment size indicating gene editing events: ~177 and ~163 bp.

(**c**) Sequencing traces of WT HL-60 cells and homozygous FES^{KO} HL-60 clone. Double traces indicate different gene editing events in the two independent FES alleles and were decomposed using TIDE analysis (https://tide.deskgen.com).

(**d**) Validation of FES knockout by immunoblot analysis on WT or FES^{KO} HL-60 cell lysate using anti-FES antibody.

(**e**) Differentiation of HL-60 cells into neutrophils is unaffected in FES^{KO} cells compared to WT or FES^{S700C} cells. CD11b surface expression prior to and after differentiation with ATRA and DMSO (1 µM and 1.25%, respectively), analyzed by flow cytometry. Threshold for CD11b-positive cells was determined using isotype control antibody.

(**f**) Analysis of putative sgRNA off-target site. Analysis of the only predicted coding off-target of the sgRNA target sequence employed for FES knockout, located in the *TCIRG1* gene. Genomic region surrounding putative off-target site was amplified by PCR, followed by Sanger sequencing analysis. No off-target gene editing events were observed. Data represent means (n = 2). Statistical analysis was performed using ANOVA with Holm-Sidak's multiple comparisons correction, NS if $P > 0.05$. Source data are provided as a Source Data file.

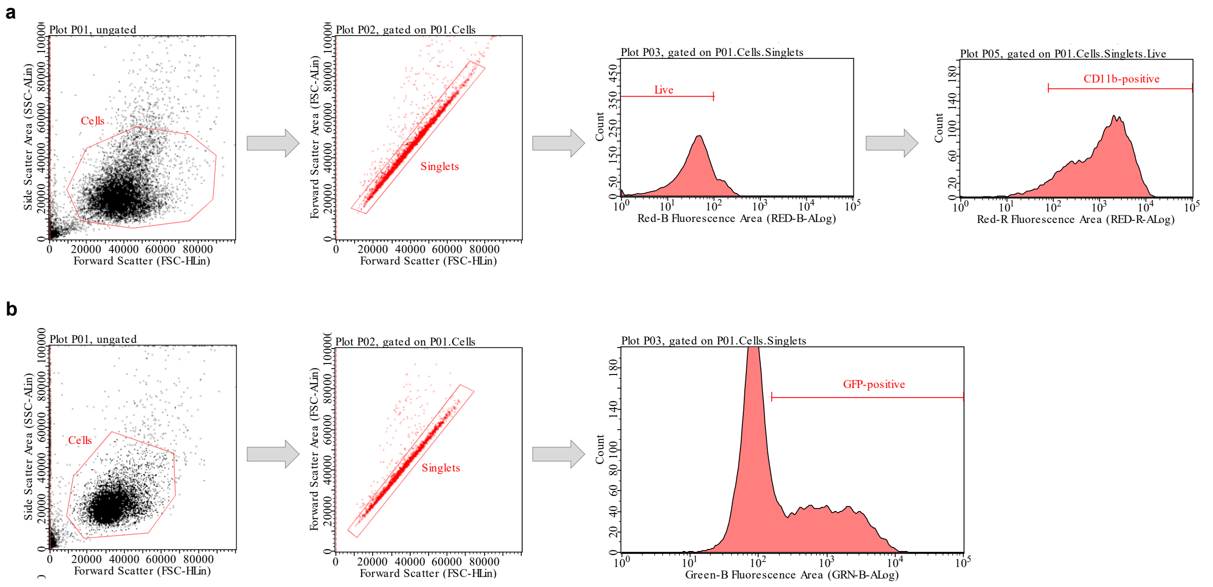

Supplementary Figure 19. Exemplary gating strategy for flow cytometry analyses.
(**a**) Gating strategy for measuring surface expression of differentiation marker CD11b on HL-60 cells. Events were gated by forward and side scatter (cells), side scatter area (singlets) and viability (live cells, 7-AAD, RED-B channel) and the threshold for CD11b-positive cells was determined based on background fluorescence for isotype control antibody and non-differentiated cells (CD11b-positive, CD11b-APC, RED-R channel). This gating strategy applies to Fig. 5e and 6c-d and Supplementary Fig. 12c and 18e.
(**b**) Gating strategy for phagocytosis assays of HL-60 neutrophils. Events were gated by forward and side scatter (cells), side scatter area (singlets) and the threshold for GFP-positive cells was determined based on background fluorescence for non-infected cells (GFP-positive, GFP, GREEN-B channel). This gating strategy applies to Fig. 7d-e and Supplementary Fig. 15.

Supplementary Tables

Supplementary Table 1. Substrate identification using PamChip® activity assay.

Substrate	Peptide	log2 of signal intensity FES^{WT}	log2 of signal intensity FES^{S700C}
CD79A_181_193	EYEDENL**Y**EGLNL	14,59	14,18
ENOG_37_49	SGASTGI**Y**EALEL	14,39	14,13
ZAP70_313_325	SVYESP**Y**SDPEEL	12,83	12,51
EFS_246_258	GGTDEGI**Y**DVPLL	12,68	12,43
PLCG1_764_776	IGTAEPD**Y**GALYE	12,56	12,48
RET_1022_1034	TPSDSLI**Y**DDGLS	12,53	12,29
IRS2_626_638	HPYPED**Y**GDIEIG	12,38	12,20
EPHA1_774_786	LDDFDGT**Y**ETQGG	12,35	12,24
PGFRB_572_584	VSSDGHE**Y**IYVDP	12,33	12,09
P85A_600_612	NENTEDQ**Y**SLVED	12,27	12,19
LAT_249_261	EEGAPD**Y**ENLQEL	12,14	12,23
PLCG2_1191_1203_C1200S	ESEEEL**Y**SSSRQL	12,14	12,04
PDPK1_2_14	ARTTSQL**Y**DAVPI	12,10	11,97
FRK_380_392	KVDNEDI**Y**ESRHE	11,99	11,88
PTN11_57_67	QNTGD**YY**DLYG	11,89	11,89
PTN11_580_590	SARV**Y**ENVGLM	11,71	11,71
KIT_930_942_C942S	ESTNHI**Y**SNLANS	11,68	11,59
PDPK1_369_381	DEDCYGN**Y**DNLLS	11,55	11,57
EGFR_1165_1177	ISLDNPD**Y**QQDFF	11,40	11,59
PECA1_708_718	DTETV**Y**SEVRK	11,36	11,53
PGFRB_709_721	RPPSAEL**Y**SNALP	11,35	11,53
EPHA7_607_619	TYIDPET**Y**EDPNR	11,30	11,43
VGFR2_989_1001	EEAPEDL**Y**KDFLT	11,26	11,38
PGFRB_1002_1014	LDTSSVL**Y**TAVQP	11,25	11,33
PTN6_558_570	KHKEDV**Y**ENLHTK	11,18	11,16
EPHA2_765_777	EDDPEAT**Y**TTSGG	11,16	11,32
PTN6_531_541	GQESE**Y**GNITY	11,15	11,29
FES_706_718	REEADGV**Y**AASGG	11,07	11,21
FER_707_719	RQEDGGV**Y**SSSGL	10,98	11,13
IRS1_890_902	PKSPGE**Y**VNIEFG	10,93	11,11

Top 30 of peptides with highest signal intensity are shown. Numbers behind substrate names indicate the amino acid residue numbers within the corresponding protein sequence. Predicted phosphorylated tyrosine residues are indicated in bold.

Supplementary Table 2. SH2 binding partner identification using PamChip® binding assay.

Binding partner	Peptide	log2 of signal intensity FES^{WT}	log2 of signal intensity FES^{S700C}
PGFRB_572_584	VSSDGHEYIYVDP	12,44	13,06
PGFRB_1014_1028	PNEGDNDYIIPLPDP	12,17	12,94
LAT_249_261	EEGAPDYENLQEL	10,79	12,19
VGFR2_1168_1180	AQQDGKDYIVLPI	10,69	11,64
ENOG_37_49	SGASTGIYEALEL	10,67	12,29
PTN11_57_67	QNTGDYYDLYG	10,66	12,05
CD3E_182_194	PVPNPDYEPIRKG	10,03	11,48
CD79A_181_193	EYEDENLYEGLNL	10,02	12,05
IRS1_890_902	PKSPGEYVNIEFG	9,97	11,28
LAT_194_206	MESIDDYVNVPES	9,51	11,14
MAPK3_198_210_C203S	ALQTPSYTPYYVA	9,32	10,68
MK12_180_189_M182B	SEBTGYVVTR	9,16	10,58
PTN6_558_570	KHKEDVYENLHTK	8,74	10,88
FGFR2_762_774	TLTTNEEYLDLSQ	8,26	9,86
TYK2_1048_1060	VPEGHEYYRVRED	7,88	9,60
JAK3_974_986	LPLDKDYYVVREP	7,87	9,77
RON_1346_1358	SALLGDHYVQLPA	7,75	9,04
FGFR3_753_765	TVTSTDEYLDLSA	7,66	9,57
MET_1227_1239	RDMYDKEYYSVHN	7,59	9,42
PTN11_580_590	SARVYENVGLM	7,27	9,94
EGFR_1190_1202	STAENAEYLRVAP	7,12	9,01
MK14_173_185	RHTDDEMTGYVAT	7,08	9,11
FAK2_572_584	RYIEDEDYYKASV	7,00	9,11
JAK1_1027_1039	AIETDKEYYTVKD	6,53	8,78
MK03_199_208	GFLTEYVATR	6,49	8,31
EPOR_419_431	ASAASFEYTILDP	6,42	7,97
MK12_178_190	ADSEMTGYVVTRW	6,15	8,13
41_654_666	LDGENIYIRHSNL	6,08	8,02
MK07_212_224	AEHQYFMTEYVAT	4,96	6,25
HAVR2_257_267	GIRSEENIYTI	4,95	4,67

Top 30 of peptides with highest signal intensity are shown. Numbers behind substrate names indicate the amino acid residues within the corresponding protein.

Supplementary Table 3. Inhibitory potency of WEL028 against a representative selection of kinases with >50 % inhibition at 1 μM in initial single-dose screen.

Kinase	Molecular weight (kDa)	Native cysteine	pIC_{50}	Apparent fold selectivity
ALK	176.4	None	6.9 ± 0.06	28
AURKA	45.8	None	6.2 ± 0.06	155
BRAF	84.4	Hinge2	7.0 ± 0.06	25
CLK4	57.5	None	5.9 ± 1.3	272
FES^{S700C}	93.5	DFG-1	8.4 ± 0.03	N/A
FLT3	112.9	DFG-1, Hinge2	5.5 ± 0.10	718
FLT4 (VEGFR3)	152.8	DFG-1, Hinge2	6.7 ± 0.07	50
GAK	143.2	DFG-1, Hinge2	7.9 ± 0.04	3
KDR (VEGFR2)	151.5	DFG-1, Hinge2	6.2 ± 0.06	153
LRRK2	286.1	None	7.3 ± 0.05	12
MAP2K1 (MEK1)	43.4	DFG-1, GK-1	6.6 ± 0.06	60
MAP2K2 (MEK2)	44.4	DFG-1, GK-1	6.6 ± 0.06	54
MAP2K4 (MEK4)	44.3	DFG-1	8.4 ± 0.03	1
MAP2K6 (MEK6)	37.5	DFG-1, GK-1	6.5 ± 0.07	68
MAP3K8 (COT)	52.9	DFG+1	6.6 ± 0.05	57
MAPKAPK5 (PRAK)	54.2	DFG-1	6.3 ± 0.09	112
MKNK2 (MNK2)	51.9	DFG-1	7.0 ± 0.06	23
PDGFRA	122.7	DFG-1, Hinge2	6.1 ± 0.06	184
PLK4	109	Hinge2	7.6 ± 0.05	6
PTK2 (FAK)	119.2	Hinge2	6.4 ± 0.06	89
ROS1	263.9	None	< 5	> 238
STK33	57.8	Hinge2	6.0 ± 0.58	254
TNK2 (ACK)	114.6	None	6.5 ± 0.26	76
TTK	97.1	GK+2	6.2 ± 0.51	152

All data (pIC_{50} ± SD, n = 2) were obtained from SelectScreen™ selectivity profiling service except for FES^{S700C}, which was determined in-house. A representative selection of kinases exhibiting >50% inhibition at 1 μM in an initial screen on 380 kinases (Supplementary Fig. 4) were selected for dose-response experiments. Assays were performed with 1 h preincubation. Indicated molecular weight is based on UniProt database records. Location of native cysteine residues in the kinase active site is indicated if applicable, with nomenclature as previously described.[1] Apparent fold selectivity was calculated as IC_{50} on that kinase divided by IC_{50} on FES^{S700C}. Source data are provided as a Source Data file.

Supplementary Table 4. Inhibitory potency of synthesized TAE684 derivatives against FER^{WT} and FER^{S701C}.

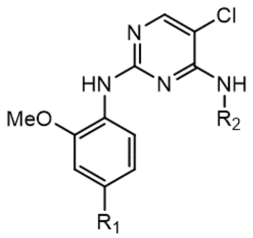

Compound	R_1	R_2	pIC_{50} FER^{WT}	pIC_{50} FER^{S700C}	Apparent fold selectivity
**1** (TAE684)	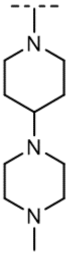	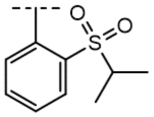	9.0 ± 0.06	8.8 ± 0.06	0,54
**2**	H	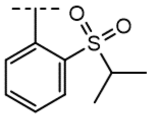	8.3 ± 0.07	8.3 ± 0.07	0,92
**4**	H	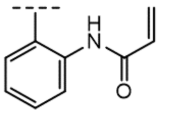	6.7 ± 0.08	8.6 ± 0.05	81
**5**	H	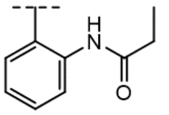	6.9 ± 0.08	7.4 ± 0.08	3,2
**6** (WEL028)	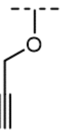	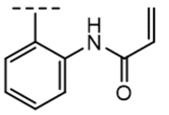	6.3 ± 0.06	8.2 ± 0.06	74

Half maximal inhibitory concentrations (expressed as pIC_{50}) were determined using recombinantly expressed FER^{WT} and FER^{S701C} in a TR-FRET assay. Final ATP concentration was 12 μM and 1 μM for FER^{WT} and FER^{S701C}, respectively. Apparent fold selectivity was calculated as IC_{50} on FER^{WT} divided by IC_{50} on FER^{S701C}. Data represent means ± SEM (n = 3). ND: not determined. Dose-response curves can be found in Supplementary Fig. 8. Source data are provided as a Source Data file.

Supplementary Table 5. List of putative off-target cleavage sites for sgRNA employed in CRISPR/Cas9-mediated mutagenesis of FES.

Mismatches	0	1	2	3
Coding	0	0	1	0
Non-coding	0	0	1	12

Potential off-target sequence	PAM	Similarity	Mismatch	Gene	Locus
TGGCTTTGGGATGTCCCGTG	*AGG*	*5*	*3,19*	*Yes*	*chr9@ 127716868-127716891*
TGACTTTGGCATGTCCTGAG	AGG	3	10,17	No	chr16@ 50371486-50371509
TTAGTTTGGGATGTCCAGAG	GGG	1	2,4,17	No	chr2@ 147874374-147874397
TGCCTTTGGTATGTCCAGAG	TAG	1	3,10,17	No	chr13@ 25630797-25630820
AGACTTTGGGAGGTCCCGTG	CAG	1	1,12,19	No	chr12@ 11447626-11447649
TGGCTGTGGGATGTCCCCAG	GAG	0	3,6,18	No	chr3@ 13295903-13295926
TGAGTCTGGGATGTCCCTAG	AGG	0	4,6,18	No	chr5@ 137202156-137202179
TGGCTTTGGGATGTCGGGAG	AAG	0	3,16,17	No	chr20@ 61773235-61773258
TGAATTTGGGATGTCCCATG	TAG	0	4,18,19	No	chr5@ 135589616-135589639
TGAATTTGGGATGCCCTGAG	AGG	0	4,14,17	No	chr5@ 61639561-61639584
TGACGTTGGGATGACCAGAG	CAG	0	5,14,17	No	chr19@ 35077359-35077382
TGACTTTGGGTTGTCCCCAT	GAG	0	11,18,20	No	chrX@ 112142232-112142255
TGACTTTGGGGTCTCCCAAG	AGG	0	11,13,18	No	chr2@ 95278649-95278672

Specificity of sgRNA was assessed using DESKGEN™ online web tool (www.deskgen.com). Sites with 3 or less mismatches compared to the sgRNA target were included. Only 1 putative off-target is located in the coding region of a gene (shown in italics).

Supplementary Table 6. List of putative off-target cleavage sites for sgRNA employed in CRISPR/Cas9-mediated knockout of FES.

Potential off-target sequence	PAM	Similarity	Mismatch	Gene	Locus
GCAGCTGCTGCAGGGCCCCG	*AGG*	*1*	*4,5,15*	*Yes*	*chr11@ 68043858-68043881*
GCATTTGATGCAGGACCCCT	CAG	4	8,20	No	chr10@ 123647319-123647342
GGCTTTGCTCCAGGACCCCG	AGG	2	2,3,10	No	chr15@ 73308252-73308275
GCAGTTACTCCAGGACCCCG	AAG	2	4,7,10	No	chr11@ 124673324-124673347
GCCCTTGCTGCAGGACCCCC	AAG	1	3,4,20	No	chr17@ 40461935-40461958
GCAGCTGCTGCAGGACCCCT	GAG	1	4,5,20	No	chr15@ 88681640-88681663
AGATTTGCTGCAGGACCCTG	AAG	1	1,2,19	No	chr10@ 127843251-127843274
GCATTTACTTCTGGACCCCG	GGG	1	7,10,12	No	chr10@ 119366165-119366188
CCATTTGCTGCTGGACCCCA	GAG	1	1,12,20	No	chr17@ 49938799-49938822
GCACTTCCTGCAGGACCCTG	GGG	1	4,7,19	No	chr11@ 1161797-1161820
GCAATTACTGCAGGACCCAG	CAG	1	4,7,19	No	chr3@ 14607567-14607590
GCAGTTGCTGCTGGACCCAG	GGG	1	4,12,19	No	chr10@ 117925154-117925177
GGATTTGCTGCAGGACTCTG	GAG	0	2,17,19	No	chr19@ 58096538-58096561
GCACTTGCTGCAGGACTCTG	CAG	0	4,17,19	No	chr4@ 173717653-173717676
ACATTTGCTGCAGGCCCCAG	AGG	0	1,15,19	No	chr5@ 144567565-144567588
GGATTTGCTGCAGGTCCCTG	CAG	0	2,15,19	No	chr18@ 46311931-46311954
TCATTTGCTGCAGGAACCCT	GAG	0	1,16,20	No	chr2@ 240787656-240787679
GCATTTACTGCATGACCCTG	GGG	0	7,13,19	No	chr16@ 59837242-59837265
GCATTTACTGCAGGACTCAG	GGG	0	7,17,19	No	chr14@ 98430008-98430031
GCATTTGCTGGAGGACTCCA	GAG	0	11,17,20	No	chr14@ 64605317-64605340
GCATTCGCTGCAGGACTCTG	CAG	0	6,17,19	No	chr6@ 32495339-32495362
GCATTTGCAGCAGGTCCCAG	GAG	0	9,15,19	No	chr20@ 63162513-63162536
GCATTTGCTGCCGGAGCCCC	AAG	0	12,16,20	No	chr16@ 1264116-1264139

Specificity of sgRNA was assessed using DESKGEN™ online web tool (www.deskgen.com). Sites with 3 or less mismatches compared to the sgRNA target were included. Only 1 putative off-target is located in the coding region of a gene (shown in italics).

Supplementary Table 7. Complete list of oligonucleotide sequences.

ID	Application	Name	Sequence
P1	Cloning	FES_SH2-KD_forw	AGGGCGCCATGGGGATTCCGGAGGTGCAGAAGC
P2	Cloning	FES_SH2-KD_rev	CACTCGAGCACCGCGGCCGCTTACCGATGCCGCT TTCGGAT
P3	Cloning	FES_I567C_forw	GTGTTGGGTGAGCAGTGTGGACGGGGGAACTTT
P4	Cloning	FES_G570C_forw	GAGCAGATTGGACGGTGCAACTTTGGCGAAGTG
P5	Cloning	FES_V575C_forw	GGGAACTTTGGCGAATGCTTCAGCGGACGCCTG
P6	Cloning	FES_L638C_forw	TACATCGTCATGGAGTGTGTGCAGGGGGGCGAC
P7	Cloning	FES_G642C_forw	GAGCTTGTGCAGGGGTGCGACTTCCTGACCTTC
P8	Cloning	FES_T646C_forw	GGGGGCGACTTCCTGTGCTTCCTCCGCACGGAG
P9	Cloning	FES_N688C_forw	GACCTGGCTGCTCGGTGCTGCCTGGTGACAGAG
P10	Cloning	FES_L690C_forw	GCTGCTCGGAACTGCTGCGTGACAGAGAAGAAT
P11	Cloning	FES_S700C_forw	AATGTCCTGAAGATCTGTGACTTTGGGATGTCC
P12	Cloning	FES_forw	CTTAAGCTTTGGTACCGCCGCCACCATGGGCTTCT CTTCTGAGC
P13	Cloning	FES_rev	CATTCTAGATCACTCGAGACCGGTCCGATGCCGC TTTCGGAT
P14	Cloning	FES_K590E_forw	ACCCTGGTGGCGGTGGAGTCTTGTCGAGAGACG
P15	Cloning	FES_K590E_rev	CGTCTCTCGACAAGACTCCACCGCCACCAGGGT
P16	Cloning	FER_SH2-KD_forw	CGTCTCCCATGATCTCCATCAGTGAGAAGCCTT
P17	Cloning	FER_SH2-KD_rev	CACCGCGGCCGCTTATGTGAGTTTTCTCTTGAT
P18	Cloning	FER_S701C_forw	AATGTTCTGAAAATCTGTGACTTTGGAATGT
P19	Cloning	FER_S701C_rev	ACATTCCAAAGTCACAGATTTTCAGAACATT
P20	Cloning	FER_forw	AGCCGTCTCGGTACCGCCGCCACCATGGGGTTTG GGAGTGACC
P21	Cloning	FER_rev	CATTCTAGATCACTCGAGACCGGTTGTGAGTTTTC TCTTGA
P22	Cloning	LYN_A384C_forw	GTCACTCATGTGCAAGATCTGTGATTTTGGCCTTG CT
P23	Cloning	LYN_A384C_rev	AGCAAGGCCAAAATCACAGATCTTGCACATGAGTG AC
P24	Cloning	PTK2_G563C_forw	GATTGTGTAAAATTATGCGACTTTGGTCTCTCCCG ATATATGGAA
P25	Cloning	PTK2_G563C_rev	TTCCATATATCGGGAGAGACCAAAGTCGCATAATT TTACACAATC
P26	Cloning	PAK4_forw	CTTAAGCTTTGGTACCGCCGCCACCATGTTTGGGA AGAGGAAGAA
P27	Cloning	PAK4_rev	CATTCTAGATCACTCGAGACCGGTTCTGGTGCGG TTCTGGCGCA
P28	Cloning	PAK4_S457C_forw	GGCAGGGTGAAGCTGTGTGACTTTGGGTTCTGC
P29	Cloning	PAK4_S457C_rev	GCAGAACCCAAAGTCACACAGCTTCACCCTGCC
P30	CRISPR mutagenesis	sgRNA_hFES-MUT_S700C_top	CACCTGACTTTGGGATGTCCCGAG
P31	CRISPR mutagenesis	sgRNA_hFES-MUT_S700C_bott	AAACCTCGGGACATCCCAAAGTCA
P32	CRISPR mutagenesis	gPCR_hFES_S700C_forw	TTTTGTCCTTGGCTTTCCTAGA
P33	CRISPR mutagenesis	gPCR_hFES_S700C_rev	GTGCTTACCCTTCTCCACAAAC
P34	CRISPR off-target analysis	HDR-template_hFES_S700C	ACTGTTGGCCAAATGAGCCCCTGCCCTGTCTCAC CCAGGGACCTGGCTGCTCGGAACTGCCTGGTGAC AGAGAAGAATGTGCTGAAGATCTGTGACTTTGGCA TGTCCCGAGAAGAAGCCGATGGGGTCTATGCAGC CTCAGGGGGCCTCAGACAAGTCCCCGTGAAGTGG ACCGCACCTGAGGCCCTTAACTA
P35	CRISPR off-target	gPCR_TTC16_forw	AGAACAGACGGTGTTGTAAGCAT
P36	CRISPR off-target	gPCR_TTC16_rev	ATTAGACAGTTGAGTTCACTGAGGC
P37	CRISPR knockout	sgRNA_hFES-KO_exon1_top	CACCGCATTTGCTGCAGGACCCCG
P38	CRISPR knockout	sgRNA_hFES-KO_exon1_bott	AAACCGGGGTCCTGCAGCAAATGC
P39	CRISPR knockout	gPCR_hFES-KO_exon1_forw	CAGTCCATCCTGACCCTACAGT
P40	CRISPR knockout	gPCR_hFES-KO_exon1_rev	AGAGTCCCATAGAGACCCACCT
P41	CRISPR off-target	gPCR_TCIRG1_forw	AGAGTCTCGTAGCTGTGTCCTTCT
P42	CRISPR off-target	gPCR_TCIRG1_rev	CAGGTACACGGCCTTCATCT

Supplementary Table 8. PLGS (v3.0.3) data processing parameters for chemical proteomics.

Parameter	Value
Lock mass m/z	785.8426
Low energy threshold	150 counts
Elevated energy threshold	30 counts
Digest reagent	trypsin
Max missed cleavages	2
Modifications	Fixed carbamidomethyl C, variable oxidation M
FDR less than	1%
Fragments/peptide	2
Fragments/protein	5
Peptides/protein	1

Supplementary Table 9. ISOQuant (v1.5) data processing parameters for chemical proteomics.

Parameter	Value
isoquant.pluginQueue.name	design project and run ISOQuant analysis
process.peptide.deplete.PEP_FRAG_2	false
process.peptide.deplete.CURATED_0	false
process.peptide.statistics.doSequenceSearch	false
process.emrt.minIntensity	1000
process.emrt.minMass	500
process.emrt.rt.alignment.match.maxDeltaMass.ppm	10
process.emrt.rt.alignment.match.maxDeltaDriftTime	2
process.emrt.rt.alignment.normalizeReferenceTime	false
process.emrt.rt.alignment.maxProcesses	24
process.emrt.rt.alignment.referenceRun.selectionMethod	AUTO
process.emrt.clustering.preclustering.orderSequence	MTMTMT
process.emrt.clustering.preclustering.maxDistance.mass.ppm	6.06E-6
process.emrt.clustering.preclustering.maxDistance.time.min	0,202
process.emrt.clustering.preclustering.maxDistance.drift	2,02
process.emrt.clustering.distance.unit.mass.ppm	6.0E-6
process.emrt.clustering.distance.unit.time.min	0,2
process.emrt.clustering.distance.unit.drift.bin	2
process.emrt.clustering.dbscan.minNeighborCount	1
process.identification.peptide.minReplicationRate	2
process.identification.peptide.minScore	6
process.identification.peptide.minOverallMaxScore	6
process.identification.peptide.minSequenceLength	6
process.identification.peptide.acceptType.PEP_FRAG_1	true
process.identification.peptide.acceptType.IN_SOURCE	false
process.identification.peptide.acceptType.MISSING_CLEAVAGE	false
process.identification.peptide.acceptType.NEUTRAL_LOSS_H20	false
process.identification.peptide.acceptType.NEUTRAL_LOSS_NH3	false
process.identification.peptide.acceptType.PEP_FRAG_2	false
process.identification.peptide.acceptType.DDA	true
process.identification.peptide.acceptType.VAR_MOD	true
process.identification.peptide.acceptType.PTM	true
process.annotation.peptide.maxSequencesPerEMRTCluster	1
process.annotation.protein.resolveHomology	true
process.annotation.peptide.maxFDR	0,01
process.annotation.useSharedPeptides	all
process.normalization.lowess.bandwidth	0,3
process.normalization.orderSequence	XPIR
process.normalization.minIntensity	3000
process.quantification.peptide.minMaxScorePerCluster	6
process.quantification.peptide.acceptType.IN_SOURCE	false
process.quantification.peptide.acceptType.MISSING_CLEAVAGE	false
process.quantification.peptide.acceptType.NEUTRAL_LOSS_H20	false
process.quantification.peptide.acceptType.NEUTRAL_LOSS_NH3	false
process.quantification.peptide.acceptType.PEP_FRAG_1	true
process.quantification.peptide.acceptType.PEP_FRAG_2	false
process.quantification.peptide.acceptType.VAR_MOD	true
process.quantification.peptide.acceptType.PTM	true
process.quantification.peptide.acceptType.DDA	true
process.quantification.topx.degree	3
process.quantification.topx.allowDifferentPeptides	true
process.quantification.minPeptidesPerProtein	2
process.quantification.absolute.standard.entry	ENO1_YEAST
process.quantification.absolute.standard.fmol	30
process.quantification.topx.allowDifferentPeptides	true
process.quantification.absolute.standard.entry	ENO1_YEAST
process.quantification.absolute.standard.fmol	30
process.quantification.maxProteinFDR	0,01

Supplementary Methods

General information

All reactions were performed using oven- or flame-dried glassware and dry solvents. Reagents were purchased from Sigma-Aldrich, Acros, and Merck and used without further purification unless noted otherwise. All moisture sensitive reactions were performed under an argon atmosphere. ^{1}H and ^{13}C NMR spectra were recorded on a Bruker AV 400 MHz spectrometer at 400.2 (^{1}H) and 100.6 (^{13}C) MHz or on a Bruker DMX-600 spectrometer at 600 (^{1}H) and 151 (^{13}C) MHz using $CDCl_3$, DMSO-*d6* or MeOD as solvent. Spectra were analyzed using MestReNova 9.1 (Mestrelab Research). Chemical shift values are reported in ppm with tetramethylsilane or solvent resonance as the internal standard ($CDCl_3$: δ 7.26 for ^{1}H, δ 77.16 for ^{13}C; DMSO-*d6*, δ 2.50 for ^{1}H, δ 39.52 for ^{13}C; MeOD: δ 3.31 for ^{1}H, δ 49.00 for ^{13}C). Data are reported as follows: chemical shifts (δ), multiplicity (s = singlet, d = doublet, dd = double doublet, td = triple doublet, t = triplet, q = quartet, br = broad, m = multiplet), coupling constants J (Hz), and integration. HPLC purification was performed on a preparative LC-MS system (Agilent 1200 serie) with an Agilent 6130 Quadruple MS detector. High-resolution mass spectra were recorded on a Thermo Scientific LTQ Orbitrap XL. Compound purity (>95% unless stated otherwise) was determined by liquid chromatography on a Finnigan Surveyor LC-MS system, equipped with a C18 column. Flash chromatography was performed using SiliCycle silica gel type SiliaFlash P60 (230−400 mesh). TLC analysis was performed on Merck silica gel 60/Kieselguhr F254, 0.25 mm. Compounds were visualized using $KMnO_4$ stain (K_2CO_3 (40 g), $KMnO_4$ (6 g) in water (600 mL)) or ninhydrin stain (ninhydrin (20 g) in ethanol (600 mL)).

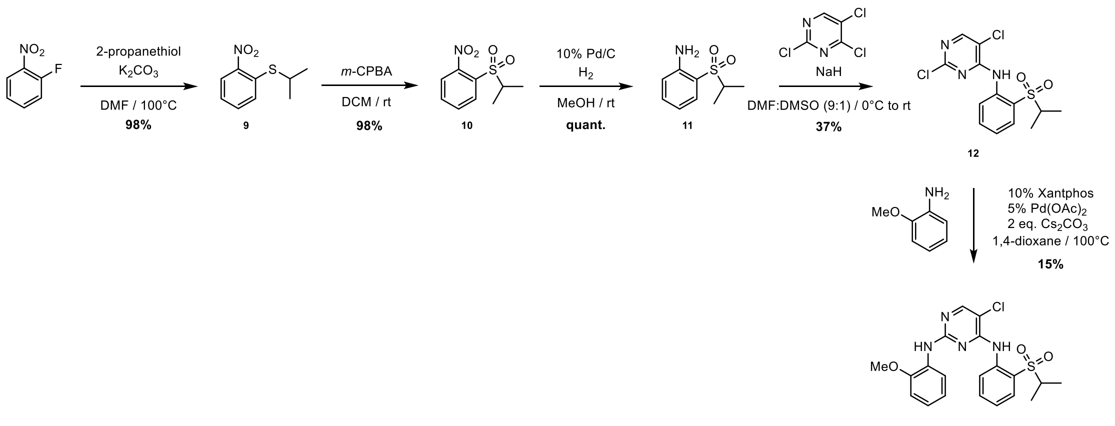

Supplementary Figure 20. Synthesis of compound 2.

*Synthesis of isopropyl(2-nitrophenyl)-sulfane (**9**)*

1-Fluoro-2-nitrobenzene (9.88 g, 70 mmol) was dissolved in dry DMF (100 mL), followed by the addition of 2-propanethiol (5.47 g, 70 mmol, 1 eq.) and potassium carbonate (24.19 g, 175 mmol, 2.5 eq.). The reaction mixture was heated at 100°C for 16 h and subsequently cooled to rt. The mixture was diluted with H_2O (200 mL) and extracted with EtOAc (3 x 200 mL). The organic layers were combined, dried over Na_2SO_4 and concentrated to obtain title compound (13.5 g, 68 mmol, 98%). ^{1}H NMR (400 MHz, $CDCl_3$): δ 8.12 (dd, *J* = 8.3, 1.5 Hz, 1H), 7.54 (ddd, *J* = 8.4, 7.1, 1.4 Hz, 1H), 7.48 (dd, *J* = 8.1, 1.4 Hz, 1H), 7.25 (ddd, *J* = 8.4, 7.1, 1.5 Hz, 1H), 3.58 (p, *J* = 6.6 Hz, 1H), 1.40 (d, *J* = 6.6 Hz, 6H). ^{13}C NMR (101 MHz, $CDCl_3$): δ 147.2, 136.5, 133.3, 128.3, 125.9, 124.9, 35.8, 22.4.

*Synthesis of 1-(isopropylsulfonyl)-2-nitrobenzene (**10**)*

Compound **9** (12.2 g, 61.3 mmol) was dissolved in DCM (120 mL), followed by portion wise addition of 3-chloroperbenzoic acid (31.6 g, 143 mmol, 2.3 eq.) The reaction mixture was stirred at rt for 16 h. The mixture was diluted with 10% Na_2SO_3 (120 mL) and stirred for 10 min, after which layers were separated and the aqueous layer was extracted with DCM (3 x 120 mL). The organic layers were combined, washed with sat. $NaHCO_3$ (2 x 200 mL), brine (1 x 250 mL), dried over $MgSO_4$ and concentrated to obtain title compound (13.9 g, 60.7 mmol, 98%). ^{1}H NMR (400 MHz, $CDCl_3$): δ 8.15 – 8.07 (m, 1H), 7.86 – 7.72 (m, 3H), 4.04-3.95 (m, 1H), 1.41 (d, *J* = 6.8 Hz, 6H). ^{13}C NMR (101 MHz, $CDCl_3$): δ 134.8, 133.1, 132.2, 125.1, 56.0, 15.5.

*Synthesis of 2-(isopropylsulfonyl)aniline (**11**)*

Compound **10** (2.03 g, 8.85 mmol) was dissolved in MeOH (25 mL), followed by addition of Pd/C (93 mg, 0.87 mmol, 0.1 eq.). The mixture was stirred under H_2 atmosphere for 16 h at rt, after which it was filtered over celite and concentrated to obtain title compound (1.76 g, 8.85 mmol, quant.). ^{1}H NMR (400 MHz, $CDCl_3$): δ 7.64 (dd, *J* = 8.0, 1.5 Hz, 1H), 7.40 – 7.30 (m, 1H), 6.80 (m, 1H), 6.74 (d, *J* = 8.2 Hz, 1H), 4.49 (s, 2H), 3.34 (m, 1H), 1.31 (d, *J* = 6.7 Hz, 6H). ^{13}C NMR (101 MHz, $CDCl_3$): δ 147.2, 135.2, 131.4, 118.3, 117.7, 117.7, 54.3, 15.4.

*Synthesis of 2,5-dichloro-N-(2-(isopropylsulfonyl)phenyl)pyrimidin-4-amine (**12**)*

A mixture of DMF/DMSO (10:1 ratio, 10 mL) was cooled to 0°C, after which NaH (354 mg, 14.8 mmol, 2.5 eq.) was added. Compound **11** (1.15 g, 5.77 mmol) was dissolved in DMF/DMSO (10:1 ratio, 5 mL) and added to the reaction mixture. The suspension was stirred at 0°C for 30 min, followed by the addition of 2,4,5-trichloropyrimidine (1.94 g, 10.6 mmol) diluted in DMF/DMSO (5 mL). The mixture was allowed to warm to rt and was then stirred for 16 h. The reaction mixture was diluted with H_2O (150 mL) and extracted with EtOAc (50 mL). The organic layer was washed with H_2O (5 x 50 mL), 5% LiCl (50 mL) and brine (50 mL), dried over $MgSO_4$ and subsequently concentrated. The crude residue was purified by flash column chromatography (pentane → 20% EtOAc in pentane), yielding title compound (777 mg, 2.24 mmol, 37%). ^{1}H NMR (400 MHz, $CDCl_3$): δ 10.06 (s, 1H), 8.62 (dd, *J* = 8.5, 1.2 Hz, 1H), 8.30 (s, 1H), 7.92 (dd, *J* = 8.0, 1.7 Hz, 1H), 7.75-7.71 (m, 1H), 7.37-7.27 (m, 1H), 3.26-3.16 (m, 1H), 1.31 (d, *J* = 6.8 Hz, 6H). ^{13}C NMR (101 MHz, $CDCl_3$): δ 157.9, 156.4, 155.7, 137.5, 135.3, 131.6, 124.7, 124.3, 122.8, 115.4, 56.2, 15.5.

*Synthesis of 5-chloro-N^4-(2-(isopropylsulfonyl)phenyl)-N^2-(2-methoxyphenyl)pyrimidine-2,4-diamine (**2**)*

Compound **12** (399 mg, 1.15 mmol), *o*-anisidine (142 mg, 1.15 mmol), XantPhos (67 mg, 0.12 mmol), $Pd(OAc)_2$ (14 mg, 0.06 mmol) and Cs_2CO_3 (756 mg, 2.30 mmol) were dissolved in dry 1,4-dioxane (15 mL) under argon and the resulting mixture was heated at 100°C for 16 h. The reaction mixture was diluted with EtOAc (50 mL), filtered over celite and subsequently concentrated. The crude residue was purified by flash column chromatography (pentane → 30% EtOAc in pentane), yielding title compound (77 mg, 0.18 mmol, 15%). HRMS (ESI+) m/z: calculated for $C_{20}H_{21}ClN_4O_3S$ ([M+H]): 433.10957; found: 433.10837. ^{1}H NMR (400 MHz, DMSO): δ 9.53 (s, 1H), 8.53 (d, *J* = 8.4 Hz, 1H), 8.37 (s, 1H), 8.25 (s, 1H), 7.82 (dd, *J* = 8.0, 1.6 Hz, 1H), 7.72 (dd, *J* = 7.9, 1.5 Hz, 1H), 7.66-7.62 (m, 1H), 7.38-7.29 (m, 1H), 7.14-7.02 (m, 2H), 6.92-6.88 (m, 1H), 3.79 (s, 3H), 3.47-3.40 (m, 1H), 1.15 (d, *J* = 6.8 Hz, 6H). ^{13}C NMR (101 MHz, DMSO): δ 158.6, 155.8, 155.3, 151.3, 138.5, 135.3, 131.4, 128.5, 124.6, 124.5, 124.0, 123.9, 123.5, 120.6, 111.6, 105.1, 56.0, 55.3, 15.3.

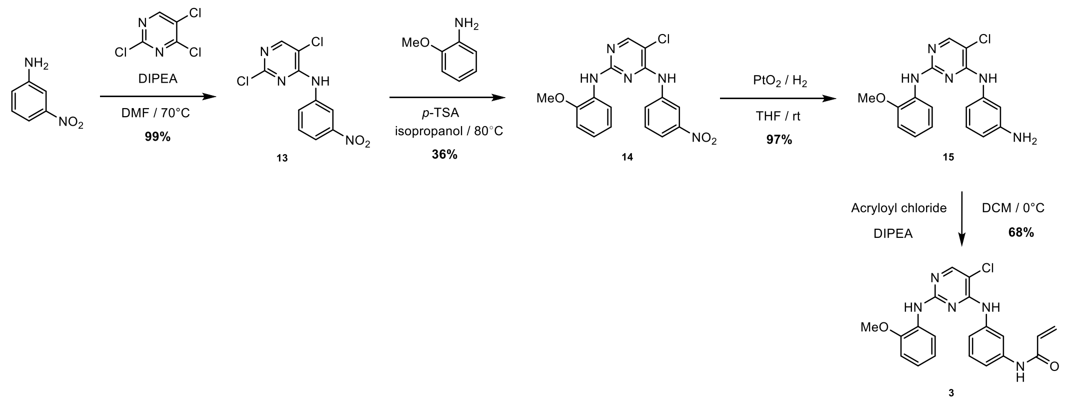

Supplementary Figure 21. Synthesis of compound 3.

*Synthesis of 2,5-dichloro-N-(3-nitrophenyl)pyrimidin-4-amine (**13**)*

2,4-5-Trichloropyrimidine (1.42 g, 7.74 mmol) and DIPEA (2.00 g, 15.5 mmol) were dissolved in DMF (4 mL). To the stirring solution was added 3-nitroaniline (1.07 g, 7.74 mmol), after which the reaction mixture was heated at reflux for 16 h. The mixture was allowed to cool down to rt and subsequently diluted with EtOAc (75 mL) and washed with H_2O (3 x 50 mL). The organic layer was dried over $MgSO_4$ and concentrated to dryness, yielding title compound (2.18 g, 7.66 mmol, 99%). ^{1}H NMR (400 MHz, DMSO): δ 9.96 (s, 1H), 8.63 (t, J = 2.2 Hz, 1H), 8.49 (s, 1H), 8.17 – 8.09 (m, 1H), 8.01 (ddd, J = 8.3, 2.3, 0.9 Hz, 1H), 7.68 (t, J = 8.2 Hz, 1H). ^{13}C NMR (101 MHz, DMSO): δ 157.0, 156.2, 147. 8, 139.0, 129.9, 129.0, 122.5, 119.1, 117.2, 114.2.

*Synthesis of 5-chloro-N^2-(2-methoxyphenyl)-N^4-(3-nitrophenyl)pyrimidine-2,4-diamine (**14**)*

Compound **13** (600 mg, 2.10 mmol) and *o*-anisidine (259 mg, 2.10 mmol) were taken up in isopropanol (20 mL), followed by the addition of *p*-TSA (400 mg, 2.10 mmol). The reaction mixture was heated under reflux for 16 h, after which it was concentrated under reduced pressure. The residue was taken up in saturated aqueous $NaHCO_3$ (20 mL) and the product was extracted with EtOAc (20 mL). The organic layer was concentrated under reduced pressure and the crude residue was purified by flash column chromatography (10% → 30% EtOAc in pentane), yielding title compound (284 mg, 0.38 mmol, 36%). ^{1}H NMR (400 MHz, DMSO): δ 9.31 (s, 1H), 8.51 (t, J = 2.2 Hz, 1H), 8.23 – 8.15 (m, 2H), 8.09 (s, 1H), 7.92 (ddd, J = 8.2, 2.3, 0.9 Hz, 1H), 7.81 – 7.74 (m, 1H), 7.55 (t, J = 8.2 Hz, 1H), 7.04 – 6.98 (m, 2H), 6.80 – 6.74 (m, 1H), 3.80 (s, 3H). ^{13}C NMR (101 MHz, DMSO): δ 157.8, 155.6, 155.5, 150.0, 147.7, 140.0, 129.5, 128.5, 128.1, 123.5, 121.8, 120.0, 117.8, 116.8, 110.9, 104.3, 55.6.

*Synthesis of N^4-(3-aminophenyl)-5-chloro-N^2-(2-methoxyphenyl)pyrimidine-2,4-diamine (**15**)*

Compound **14** (200 mg, 0.54 mmol) was dissolved in anhydrous THF (10 mL), followed by addition of PtO_2 (21 mg, 0.09 mmol, 0.1 eq.). The mixture was stirred under H_2 atmosphere for 72 h at rt, after which it was diluted in MeOH, filtered over celite and concentrated to obtain title compound (179 mg, 0.54 mmol, 97%), which was directly used in the next step.

*Synthesis of N-(3-((5-chloro-2-((2-methoxyphenyl)amino)pyrimidin-4-yl)amino)phenyl)acrylamide (**3**)*

Compound **15** (77 mg, 0.23 mmol) was taken up in DCM (2 mL) and cooled to 0°C. Subsequently, DIPEA (29 mg, 0.23 mmol) was added and the reaction mixture was stirred for 10 min. Acryloyl chloride (20 mg, 0.23 mmol) dissolved in DCM (1 mL) was dropwisely added to the mixture. After stirring for 30 min, the reaction was quenched by addition of water (5 mL). The product was extracted from the reaction mixture with DCM (3 x 25 mL), the organic extract was dried over $MgSO_4$ and concentrated to dryness. The crude residue was purified by flash column chromatography (10% → 40% EtOAc in pentane), yielding title compound (61 mg, 0.15 mmol, 68%). HRMS (ESI+) m/z: calculated for $C_{20}H_{18}ClN_5O_2$ ([M+H]): 396.12218; found: 396.12156. ^{1}H NMR (400 MHz, $CDCl_3$): δ 8.21 (dd, *J* = 7.9, 1.6 Hz, 1H), 8.04 (d, *J* = 12.2 Hz, 2H), 7.70 (s, 1H), 7.58 (d, *J* = 7.9 Hz, 1H), 7.41 (s, 1H), 7.33 (t, *J* = 8.0 Hz, 1H), 7.26 (d, *J* = 8.5 Hz, 1H), 7.16 (s, 1H), 7.04 – 6.95 (m, 1H), 6.94 – 6.83 (m, 2H), 6.45 (dd, *J* = 16.9, 1.3 Hz, 1H), 6.23 (dd, *J* = 16.8, 10.2 Hz, 1H), 5.79 (dd, *J* = 10.2, 1.3 Hz, 1H), 3.88 (s, 3H). ^{13}C NMR (101 MHz, $CDCl_3$): δ 155.7, 148.8, 138.6, 138.5, 131.2, 129.6, 128.8, 128.2, 122.6, 120.5, 120.1, 117.3, 116.5, 115.7, 112.8, 110.3, 105.1, 55.8.

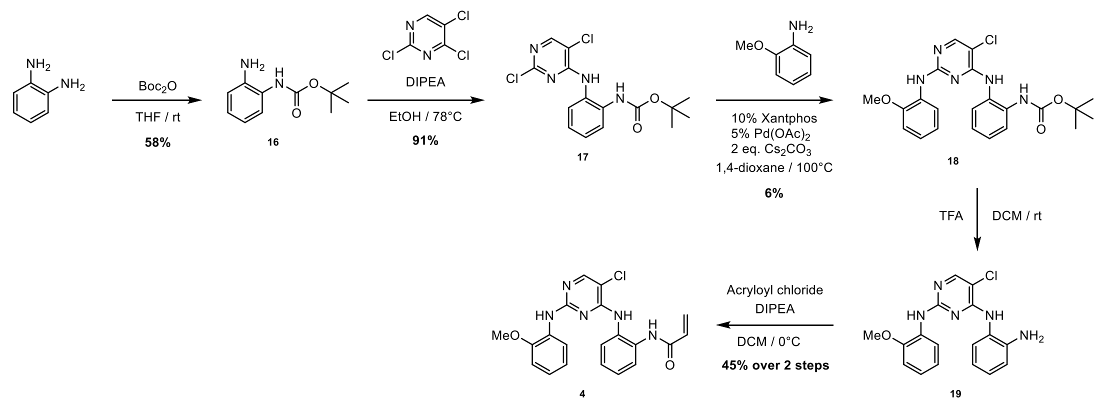

Supplementary Figure 22. Synthesis of compound 4.

*Synthesis of tert-butyl (2-aminophenyl)carbamate (**16**)*

To a solution of *o*-phenylenediamine (2.19 g, 20.2 mmol) in THF (20 mL) was dropwisely added a solution of $(Boc)_2O$ (4.45 g, 20.4 mmol) in THF (5 mL). The reaction mixture was stirred at rt for 16 h, after which the mixture was concentrated under reduced pressure and the residue was taken up in a cold mixture of EtOAc/Petroleum ether (1:4 ratio, 15 mL), causing the product to precipitate. The precipitate was collected by filtration and dried to yield the title compound (2.43 g, 11.7 mmol, 58%). ^1H NMR (400 MHz, $CDCl_3$): δ 7.28 (s, 1H), 7.00 (td, *J* = 7.6, 1.5 Hz, 1H), 6.82 – 6.74 (m, 2H), 6.28 (s, 1H), 3.55 (s, 2H), 1.51 (s, 9H). ^{13}C NMR (101 MHz, $CDCl_3$): δ 154.0, 140.0, 126.3, 124.9, 120.5, 119.7, 117.7, 117.0, 80.6, 28.5, 28.4. Spectroscopic data are in accordance with those reported in literature[2].

*Synthesis of tert-butyl (2-((2,5-dichloropyrimidin-4-yl)amino)phenyl)carbamate (**17**)*

2,4-5-Trichloropyrimidine (1.18 g, 6.43 mmol) and DIPEA (1.68 g, 13.0 mmol) were dissolved in EtOH (25 mL). To the stirring solution was added compound **16** (1.35 g, 6.48 mmol), after which the reaction mixture was heated at reflux for 16 h. After TLC indicated depletion of starting material, the mixture was allowed to cool down to rt and subsequently triturated with cold H_2O (20 mL), causing precipitation of the product. The precipitate was collected by vacuum filtration and dried to yield the title compound (2.11 g, 5.94 mmol, 92%). ^1H NMR (400 MHz, $CDCl_3$): δ 8.64 (s, 1H), 8.16 (s, 1H), 7.78 (dd, *J* = 8.1, 1.2 Hz, 1H), 7.32-7.27 (m, 1H), 7.24 – 7.16 (m, 2H), 6.59 (s, 1H), 1.53 (s, 9H). ^{13}C NMR (101 MHz, $CDCl_3$): δ 158.3, 157.4, 154.8, 154.7, 130.9, 130.2, 126.6, 126.5, 126.4, 124.9, 114.4, 82.0, 28.4.

*Synthesis of tert-butyl(2-((5-chloro-2-((2-methoxyphenyl)amino)pyrimidin-4-yl)amino)phenyl)carbamate (**18**)*

Compound **17** (600 mg, 1.69 mmol), *o*-anisidine (208 mg, 1.69 mmol), Xantphos (98 mg, 0.17 mmol), $Pd(OAc)_2$ (19 mg, 0.084 mmol) and cesium carbonate (1.10 g, 3.38 mmol) were dissolved in 20 mL dry 1,4-dioxane. The reaction mixture was purged with argon and subsequently heated at 100°C for 16 h. Subsequently, the mixture was diluted with EtOAc and filtered over Celite®, after which the filtrate was concentrated. The crude residue was purified by flash column chromatography (pentane → 20% EtOAc in pentane), yielding title compound (48 mg, 0.11 mmol, 6%). ^1H NMR (400 MHz, $CDCl_3$): δ 8.12

– 8.03 (m, 2H), 7.79 (s, 1H), 7.72 – 7.66 (m, 1H), 7.63 (s, 1H), 7.49 – 7.41 (m, 1H), 7.29 – 7.22 (m, 2H), 6.89 (m, 1H), 6.82 (dd, *J* = 8.1, 1.5 Hz, 1H), 6.78 – 6.70 (m, 1H), 6.66 (s, 1H), 3.85 (s, 3H), 1.50 (s, 9H). ^{13}C NMR (101 MHz, $CDCl_3$): δ 157.0, 154.1, 147.8, 131.9, 130.9, 129.2, 128.9, 127.6, 126.5, 125.4, 123.9, 121.6, 120.8, 118.7, 114.2, 109.8, 105.2, 81.5, 55.8, 28.4.

*Synthesis of N^4-(2-aminophenyl)-5-chloro-N^2-(2-methoxyphenyl)pyrimidine-2,4-diamine (**19**)*

Compound **18** (50 mg, 0.11 mmol) was dissolved in DCM (3 mL), after which an equal volume of TFA was slowly added. The reaction mixture was stirred for 16 h at rt and subsequently evaporated to dryness to afford the title compound, which was directly used in the next reaction.

*Synthesis of N-(2-((5-chloro-2-((2-methoxyphenyl)amino)pyrimidin-4-yl)amino)phenyl)acrylamide (**4**)*

Compound **19** (84 mg, 0.18 mmol) was taken up in DCM (2 mL) and cooled to 0°C. Subsequently, DIPEA (24 mg, 0.18 mmol) was added and the reaction mixture was stirred for 10 min. Acryloyl chloride (17 mg, 0.18 mmol) dissolved in DCM (1 mL) was dropwisely added to the mixture. After stirring for 15 min, the reaction was quenched by addition of water (5 mL). The product was extracted from the reaction mixture with DCM (50 mL), the organic extract was dried over $MgSO_4$ and concentrated to dryness. The crude residue was purified by flash column chromatography (10% → 40% EtOAc in pentane), yielding title compound (20 mg, 0.05 mmol, 45% over two steps). HRMS (ESI+) m/z: calculated for $C_{20}H_{18}ClN_5O_2$ ([M+H]): 396.12218; found: 396.12156. ^{1}H NMR (400 MHz, MeOD): δ 8.04 (s, 1H), 7.81 (dd, *J* = 8.1, 1.6 Hz, 1H), 7.76 – 7.69 (m, 1H), 7.51 – 7.44 (m, 1H), 7.41 – 7.33 (m, 2H), 7.03 – 6.95 (m, 2H), 6.73 (ddd, *J* = 8.6, 7.1, 1.8 Hz, 1H), 6.48 (dd, *J* = 17.0, 9.5 Hz, 1H), 6.40 (dd, *J* = 16.9, 2.4 Hz, 1H), 5.81 (dd, *J* = 9.4, 2.4 Hz, 1H), 3.87 (s, 3H). ^{13}C NMR (101 MHz, MeOD): δ 166.9, 158.8, 149.9, 132.9, 132.5, 131.4, 129.9, 129.2, 128.9, 128.2, 127.9, 127.4, 126.0, 125.5, 125.0, 122.0, 121.6, 111.5, 106.5, 56.3.

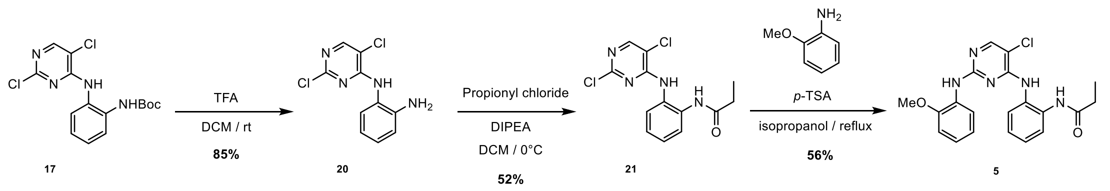

Supplementary Figure 23. Synthesis of compound 5.

*Synthesis of N[1]-(2,5-dichloropyrimidin-4-yl)benzene-1,2-diamine (**20**)*

Compound **17** (323 mg, 0.91 mmol) was dissolved in DCM (10 mL), after which TFA (3 mL) was slowly added. The rection mixture was stirred at rt for 16 h and subsequently evaporated to dryness. The residue was dissolved in water, neutralized with aqueous K_2CO_3 to pH 7-8 and the resulting precipitate was collected by filtration and dried to afford title compound (197 mg, 0.77 mmol, 85%). ^{1}H NMR (400 MHz, DMSO): δ 9.01 (s, 1H), 8.14 (s, 1H), 7.03 (d, *J* = 7.8 Hz, 1H), 6.95 (s, 1H), 6.72 (d, *J* = 8.0 Hz, 1H), 6.54 (t, *J* = 7.5 Hz, 1H), 4.92 (s, 2H). ^{13}C NMR (101 MHz, DMSO): δ 158.7, 158.2, 157.3, 154.4, 144.9, 128.4, 127.8, 121.7, 115.8, 115.6.

*Synthesis of N-(2-((2,5-dichloropyrimidin-4-yl)amino)phenyl)propionamide (**21**)*

Compound **20** (171 mg, 0.67 mmol) was dissolved in DCM (5 mL) and cooled to 0°C. Subsequently, DIPEA (85 mg, 0.67 mmol) was added and the reaction mixture was stirred for 10 min. Propionyl chloride (64 mg, 0.67 mmol) dissolved in DCM (3 mL) was dropwisely added. After stirring for 2 h, the reaction mixture was diluted with DCM (50 mL) and washed with H_2O (2 x 50 mL). The organic extracted extract was dried over $MgSO_4$ and concentrated to dryness. The crude residue was purified by flash column chromatography (20% → 30% EtOAc in pentane), yielding title compound (109 mg, 0.35 mmol, 52%). ^{1}H NMR (400 MHz, $CDCl_3$): δ 8.67 (s, 1H), 8.16 (s, 1H), 8.07 (s, 1H), 7.73 (dd, *J* = 8.2, 1.3 Hz, 1H), 7.32 – 7.23 (m, 1H), 7.14 – 7.05 (m, 1H), 6.87 (dd, *J* = 7.9, 1.5 Hz, 1H), 2.38 (q, *J* = 7.6 Hz, 2H), 1.20 (t, *J* = 7.6 Hz, 3H). ^{13}C NMR (101 MHz, $CDCl_3$): δ 174.4, 158.1, 157.4, 155.0, 131.4, 130.2, 127.0, 126.5, 126.1, 125.0, 114.7, 30.0, 10.2.

*Synthesis of N-(2-((5-chloro-2-((2-methoxyphenyl)amino)pyrimidin-4-yl)amino)phenyl)propionamide (**5**)*

Compound **21** (59 mg, 0.19 mmol) and *o*-anisidine (23 mg, 0.19 mmol) were taken up in isopropanol (10 mL), followed by the addition of *p*-TSA (37 mg, 0.19 mmol). The reaction mixture was heated under reflux for 16 h, after which it was concentrated under reduced pressure. The residue was taken up in saturated aqueous $NaHCO_3$ (20 mL) and the product was extracted with EtOAc (20 mL). The organic layer was dried over $MgSO_4$ and concentrated. The crude residue was purified by flash column chromatography (10% → 40% EtOAc in pentane), yielding a mixture of compound **21** and **5**, which was further purified by HPLC, affording title compound (42 mg, 0.10 mmol, 56%). HRMS (ESI+) m/z: calculated for $C_{20}H_{20}ClN_5O_2$ ([M+H]): 398.13783; found: 398.13722. ^{1}H NMR (400 MHz, $CDCl_3$): δ 10.04 (s, 1H), 9.24 (s, 1H), 7.85 (s, 1H), 7.68 – 7.60 (m, 2H), 7.58 (dd, *J* = 8.0, 1.6 Hz, 1H), 7.26 (s, 3H), 7.16 (dd, *J* = 7.5, 2.0 Hz, 1H), 7.06 (ddd, *J* = 8.3, 7.6, 1.6 Hz, 1H), 6.84 (dd, *J* = 8.3, 1.4 Hz, 1H), 6.70 (td, *J* = 7.8, 1.3 Hz, 1H), 3.80 (s, 3H), 2.46 (q, *J* = 7.5 Hz, 2H), 1.26 (t, *J* = 7.6 Hz, 3H). ^{13}C NMR (101 MHz,

$CDCl_3$): δ 174.3, 157.7, 153.3, 152.8, 151.2, 130.6, 127.8, 127.6, 126.9, 126.1, 125.8, 124.9, 123.1, 120.3, 111.0, 105.4, 56.0, 30.3, 10.2.

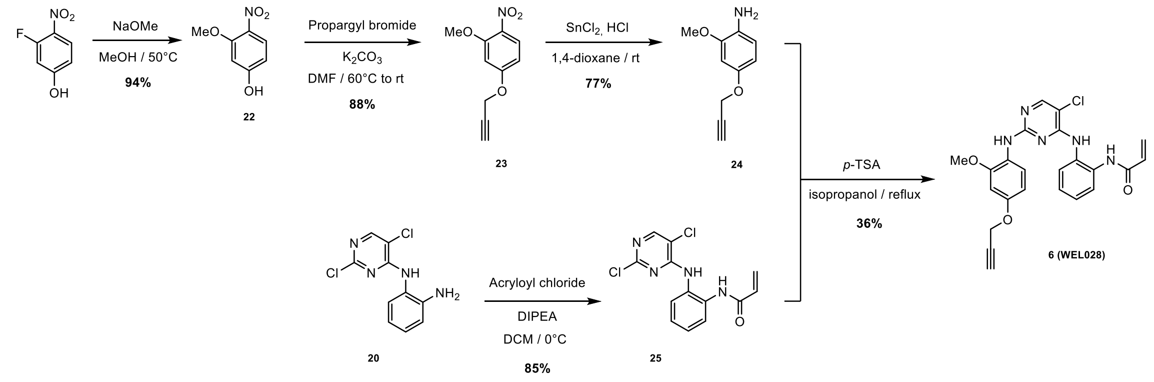

Supplementary Figure 24. Synthesis of compound 6 (WEL028).

*Synthesis of 3-methoxy-4-nitrophenol (**22**)*

3-Fluoro-4-nitrophenol (1.00 g, 6.4 mmol) was added to a solution of NaOMe in MeOH (0.5M, 14 mL), which was then heated at 50°C for 12 h. Additional NaOMe in MeOH (0.5M, 14 mL) was added and the mixture was stirred at 50°C until TLC indicated complete depletion of starting material. The mixture was diluted with H_2O (100 mL), neutralized with 3M HCl and then extracted with EtOAc (3 x 100 mL). The combined organic layers were washed with brine (100 mL), dried over $MgSO_4$ and evaporated to yield title compound (1.01 g, 6.0 mmol, 94%). ^{1}H NMR (400 MHz, DMSO): δ 10.90 (s, 1H), 7.89 (d, J = 9.0 Hz, 1H), 6.60 (d, J = 2.4 Hz, 1H), 6.47 (dd, J = 9.0, 2.4 Hz, 1H), 3.86 (s, 3H). ^{13}C NMR (101 MHz, DMSO): δ 164.4, 156.1, 131.2, 128.8, 108.0, 100.8, 56.8. Spectroscopic data are in accordance with those reported in literature[3].

*Synthesis of 2-methoxy-1-nitro-4-(prop-2-yn-1-yloxy)benzene (**23**)*

Compound **22** (600 mg, 3.6 mmol) and K_2CO_3 (1.47 g, 10.6 mmol) were taken up in anhydrous DMF (10 mL) and heated at 60°C for 30 min under argon. The reaction mixture was cooled to rt, after which propargyl bromide (1.34 g, 9.0 mmol as 80% (w/w) solution in toluene) was added. The mixture was stirred at rt for 16 h, after which it was poured into ice water (200 mL) with stirring for 10 min. The formed precipitate was collected by filtration and dried under vacuum to yield title compound (648 mg, 3.1 mmol, 88%), which was directly used in the next reaction.

*Synthesis of 2-methoxy-4-(prop-2-yn-1-yloxy)aniline (**24**)*

Compound **23** (600 mg, 2.9 mmol) was dissolved in 1,4-dioxane (10 mL) and cooled to 0°C. Cooled (0 °C) stannous chloride dihydrate (3.28 g, 14.5 mmol) in concentrated HCl (10 mL) was dropwisely added to the reaction mixture. After stirring for 16 h at rt, the mixture was basified to pH > 9 by addition of NaOH pellets and extracted with DCM (4 x 10 mL). The organic layer was washed with brine (1 x 10 mL), dried over $MgSO_4$ and concentrated to dryness. The crude product was then purified by flash column chromatography (pentane → 25% EtOAc in pentane) to afford title compound (395 mg, 2.23 mmol, 77%). ^{1}H NMR (400 MHz, $CDCl_3$): δ 6.62 (d, J = 8.4 Hz, 1H), 6.52 (d, J = 2.6 Hz, 1H), 6.42 (dd,

J = 8.5, 2.6 Hz, 1H), 4.61 (d, J = 2.4 Hz, 2H), 3.81 (s, 3H), 3.49 (s, 2H), 2.51 (t, J = 2.4 Hz, 1H). ^{13}C NMR (101 MHz, $CDCl_3$): δ 151.0, 148.3, 130.8, 115.0, 105.9, 100.7, 79.3, 75.3, 56.8, 55.6.

*Synthesis of N-(2-((2,5-dichloropyrimidin-4-yl)amino)phenyl)acrylamide (**25**)*

Compound **20** (250 mg, 0.98 mmol) was dissolved in DCM (5 mL) and cooled to 0°C. Subsequently, DIPEA (127 mg, 0.98 mmol) was added and the reaction mixture was stirred for 10 min. Acryloyl chloride (93 mg, 1.03 mmol) dissolved in DCM (1 mL) was dropwisely added to the mixture. After stirring for 1 h, the reaction was quenched by addition of water (50 mL). The mixture was extracted with DCM (50 mL), the organic extract was dried over $MgSO_4$ and concentrated. The crude residue was purified by flash column chromatography (20% → 40% EtOAc in pentane), yielding title compound (259 mg, 0.84 mmol, 85%). ^{1}H NMR (400 MHz, $CDCl_3$): δ 8.72 (d, J = 18.0 Hz, 2H), 8.13 (s, 1H), 7.72 (dd, J = 8.2, 1.3 Hz, 1H), 7.26 (td, J = 7.8, 1.5 Hz, 1H), 7.08 (td, J = 7.7, 1.4 Hz, 1H), 6.95 (dd, J = 8.0, 1.5 Hz, 1H), 6.44 (dd, J = 16.9, 1.4 Hz, 1H), 6.30 (dd, J = 16.9, 10.1 Hz, 1H), 5.77 (dd, J = 10.1, 1.6 Hz, 1H). ^{13}C NMR (101 MHz, $CDCl_3$): δ 165.2, 157.8, 157.2, 154.7, 131.1, 129.8, 129.8, 129.0, 127.0, 126.3, 125.8, 125.0, 114.6.

*Synthesis of N-(2-((5-chloro-2-((2-methoxy-4-(prop-2-yn-1-yloxy)phenyl)amino)pyrimidin-4-yl)amino)-phenyl)acrylamide (**6, WEL028**)*

Compound **24** (26 mg, 0.15 mmol) and compound **25** (46 mg, 0.15 mmol) were taken up in isopropanol (5 mL), followed by the addition of *p*-TSA (28 mg, 0.15 mmol). The reaction mixture was heated under reflux for 16 h, after which it was concentrated under reduced pressure. The residue was taken up in saturated aqueous $NaHCO_3$ (20 mL) and the product was extracted with EtOAc (20 mL). The organic layer was dried over $MgSO_4$ and concentrated to dryness. The crude residue was purified by flash column chromatography (10% → 35% EtOAc in pentane), yielding title compound (24 mg, 0.05 mmol, 36%). HRMS (ESI+) m/z: calculated for $C_{23}H_{20}ClN_5O_3$ ([M+H]): 450.13274; found: 450.13231. ^{1}H NMR (400 MHz, $CDCl_3$): δ 8.04 (d, J = 17.6 Hz, 2H), 7.87 (d, J = 8.9 Hz, 1H), 7.67 (s, 1H), 7.59 (t, J = 4.8 Hz, 2H), 7.38 (s, 1H), 7.29 (dd, J = 6.0, 3.5 Hz, 2H), 6.52 (d, J = 2.7 Hz, 1H), 6.40 – 6.31 (m, 2H), 6.16 (dd, J = 16.9, 10.3 Hz, 1H), 5.72 (d, J = 10.2 Hz, 1H), 4.65 (d, J = 2.4 Hz, 2H), 3.81 (s, 3H), 2.54 (t, J = 2.4 Hz, 1H). ^{13}C NMR (101 MHz, $CDCl_3$): δ 164.6, 157.8, 156.9, 153.5, 149.6, 131.6, 131.1, 130.8, 128.4, 127.2, 126.8, 126.4, 125.1, 123.2, 120.1, 105.1, 99.8, 78.9, 75.7, 56.5, 55.9.

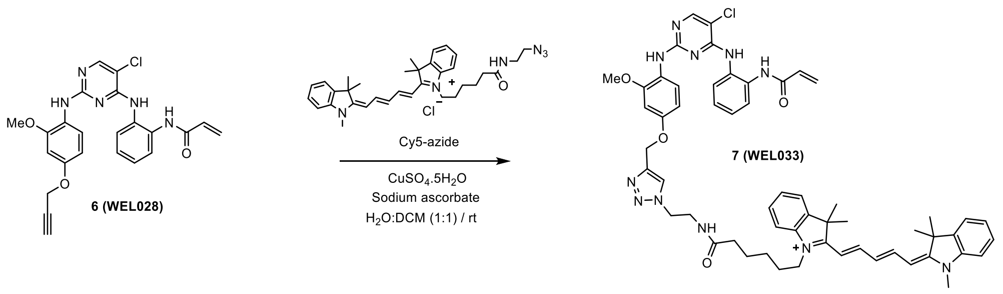

Supplementary Figure 25. Synthesis of compound 7 (WEL033).

*Synthesis of 1-(6-((2-(4-((4-((4-((2-acrylamidophenyl)amino)-5-chloropyrimidin-2-yl)amino)-3-methoxy-phenoxy)methyl)-1H-1,2,3-triazol-1-yl)ethyl)amino)-6-oxohexyl)-3,3-dimethyl-2-((1E,3E)-5-((E)-1,3,3-trimethylindolin-2-ylidene)penta-1,3-dien-1-yl)-3H-indol-1-ium (**7, WEL033**)*

Compound **6** (30 mg, 0.07 mmol) and Cy5-azide (77 mg, 0.14 mmol) were dissolved in degassed DCM (3 mL). $CuSO_4$ (8 mg, 0.01 mmol) and sodium ascorbate (16 mg, 0.03 mmol) were separately dissolved in degassed H_2O (3 mL) and added to the reaction mixture. The reaction mixture was vigorously stirred for 16 h at rt and subsequently evaporated to dryness. The crude residue was purified by flash column chromatography (DCM → 2% MeOH in DCM), followed by further HPLC purification to afford title compound (3.2 mg, 3 µmol, 5%). HRMS (ESI+) m/z: calculated for $[C_{57}H_{63}ClN_{11}O_4]^+$: 1000.47475; found: 1000.47462. ^1H NMR (600 MHz, MeOD): δ 8.20 (td, *J* = 13.1, 3.2 Hz, 2H), 8.07 (s, 1H), 7.97 (s, 1H), 7.66 (dd, *J* = 7.8, 1.7 Hz, 1H), 7.52 – 7.45 (m, 4H), 7.39 (m, 2H), 7.35 – 7.30 (m, 2H), 7.29 (d, *J* = 7.1 Hz, 1H), 7.27 – 7.22 (m, 4H), 6.66 (d, *J* = 2.6 Hz, 1H), 6.57 (t, *J* = 12.4 Hz, 1H), 6.51 – 6.42 (m, 2H), 6.40 (dd, *J* = 16.9, 1.9 Hz, 1H), 6.27 (d, *J* = 13.7 Hz, 1H), 6.19 (d, *J* = 13.7 Hz, 1H), 5.82 (dd, *J* = 9.9, 1.9 Hz, 1H), 5.17 (s, 2H), 4.57 – 4.53 (m, 2H), 4.04 (t, *J* = 7.7 Hz, 2H), 3.77 (s, 3H), 3.69 – 3.65 (m, 2H), 3.56 (s, 3H), 2.66 (s, 3H), 2.15 (t, *J* = 7.2 Hz, 2H), 1.79 – 1.58 (m, 18H), 1.40 – 1.31 (m, 2H). ^{13}C NMR (151 MHz, MeOD): δ 176.2, 175.3, 174.6, 166.9, 155.4, 145.1, 144.2, 143.5, 142.6, 142.5, 133.0, 131.4, 129.8, 129.7, 129.0, 128.7, 128.1, 127.4, 126.6, 126.2, 126.2, 126.0, 125.6, 123.4, 123.2, 112.0, 111.8, 106.8, 104.3, 104.2, 100.7, 62.8, 56.4, 49.8, 44.8, 40.3, 36.5, 28.1, 27.9, 27.9, 27.8, 27.1, 26.4.

Supplementary Note 1

Applicability of chemical genetics strategy to other kinases

To investigate the broader applicability of our strategy employing engineered kinases, we explored whether the complementary probes could also target corresponding DFG-1 cysteine mutants of kinases other than FES. To this end, the DFG-1 residue (Ser701) of the FES-related kinase FER was mutated into a cysteine. FER^{WT} and FER^{S701C} were recombinantly expressed, purified and biochemically characterized and exhibited similar affinity for ATP (K_M = 11 µM for FER^{WT} and K_M = 3.5 µM for FER^{S701C}; Supplementary Fig. 8a). Profiling the panel of synthesized compounds on FER^{WT} and FER^{S701C} (Supplementary Table 4 and Supplementary Fig. 8b-f) revealed that WEL028 potently targeted mutant but not wild-type FER (pIC_{50} = 6.3 ± 0.36 for FER^{WT}, pIC_{50} = 8.2 ± 0.06 for FER^{S701C}). Moreover, incubation of cell lysates from HEK293T cells overexpressing FER^{S701C} with WEL033 resulted in dose-dependent labeling that was prevented by preincubation with inhibitor **4** (Supplementary Fig. 9a-b). These results endorse that WEL028 is the first compound that allows acute modulation of FES activity without affecting FER.

Subsequently, we generated cysteine mutants of three other tyrosine kinases with lower sequence similarity and different amino acids at the DFG-1 position: LYN, PTK2 and PAK4 (resulting in LYN^{A384C}, $PTK2^{G536C}$ and $PAK4^{S457C}$, respectively). Overexpression of these kinases and incubation with complementary probes exhibited dose-dependent, mutant-specific labeling (Supplementary Fig. 9c-h). Of note, no labeling was observed for LYN^{A384C} with one-step probe WEL033 (data not shown), which may indicate that the bulky Cy5 fluorophore prohibits active site binding in this particular case. The chemical structure of WEL028 could clearly be further optimized to improve its potency on these mutant kinases individually. Nevertheless, these results suggest that our chemical genetics strategy employing mutagenesis of the DFG-1 residue is not exclusively applicable to FES, but is possibly also suited to visualize target engagement of other kinases.

Supplementary Discussion

Based on our experimental results, we proposed a mechanistic model for the role of FES in the activation of a SYK/HS-1/PLCγ2 pathway during neutrophil phagocytosis (Fig. 8). In this light, it is relevant to note that FES possesses an FX domain that binds phosphatidic acid (PA) and activates its kinase activity.[4] PA is synthesized by phospholipase D (PLD) in response to activation of various immune receptors. PLD activation also leads to elevated intracellular Ca^{2+} levels, but the exact mechanistic pathways underlying this event remain poorly understood.[5] FES could possibly be a molecular link that activates PLCγ2 to increase Ca^{2+} in response to PA production by PLD. Further studies are necessary to investigate the activation mechanism of FES by PA and the role of FES in the complex cross-talk between PLD and PLCγ2.

In this work, we focused on the role of FES in SYK activation during neutrophil phagocytosis. The question arises under which other circumstances FES activates SYK. Our proposed model may also be applicable to mouse macrophages, as a previous study reported a consistent reduction in bacterial phagocytosis by peritoneal macrophages from FES knock-out mice.[6] However, it was found that FES is not required for SYK, HS1 and PLCγ2 in mouse platelets stimulated with collagen.[7] This highlights that FES is activated downstream of specific (immuno)receptors and is likely to be involved only in specific pathways, which may differ among different cell types.

The identification of FES as a potential activator of SYK also provides new insights in previous studies reporting on FES and/or SYK function. For example, FES is involved in the development and function of osteoclasts, multinucleated cells responsible for bone resorption.[8] Accordingly, SYK-deficient osteoclasts exhibit major defects in the actin cytoskeleton, resulting in reduced bone resorption.[9] It remains to be determined whether FES inhibition disrupts the osteoclast cytoskeleton in a similar manner, which would make FES a potential target to treat osteoporosis and cancer-associated bone disease. Moreover, FES inhibition was shown to suppress growth of acute myeloid leukemia (AML) cells that express the FLT3-ITD mutation, but not cells expressing wild-type FLT3.[10] Similarly, FLT3-ITD AML is more vulnerable to SYK suppression than FLT3-WT AML.[11] It would be interesting to investigate whether FES activates SYK in these AML cells and whether altered internalization of FLT3-ITD compared to FLT3-WT perhaps explains the increased susceptibility to FES and SYK inhibition.

Two SYK inhibitors have recently been approved by the FDA and more are currently in clinical trials for the treatment of various malignancies and inflammatory diseases.[12] Notably, FES is expressed in many cell types that contribute to the pathogenesis of these diseases, such as macrophages, mast cells, neutrophils and B-cells, but future studies will prove whether FES inhibitors may be of therapeutic value in these disorders.

Supplementary References

1. Liu, Q. *et al.* Developing irreversible inhibitors of the protein kinase cysteinome. *Chem. Biol.* **20**, 146–159 (2013).
2. Wong, J. C. *et al.* Pharmacokinetic optimization of class-selective histone deacetylase inhibitors and identification of associated candidate predictive biomarkers of hepatocellular carcinoma tumor response. *J. Med. Chem.* **55**, 8903–8925 (2012).
3. Ménard, D. *et al.* Novel potent BRAF inhibitors: Toward 1 nM compounds through optimization of the central phenyl ring. *J. Med. Chem.* **52**, 3881–3891 (2009).
4. Di Fulvio, M. *et al.* Phospholipase D2 (PLD2) shortens the time required for myeloid leukemic cell differentiation: Mechanism of action. *J. Biol. Chem.* **287**, 393–407 (2012).
5. Nunes, P. & Demaurex, N. The role of calcium signaling in phagocytosis. *J. Leukoc. Biol.* **88**, 57–68 (2010).
6. Parsons, S. A. & Greer, P. A. The Fps/Fes kinase regulates the inflammatory response to endotoxin through down-regulation of TLR4, NF-κB activation, and TNF-α secretion in macrophages. *J. Leukoc. Biol.* **80**, 1522–1528 (2006).
7. Senis, Y. A. *et al.* Fps/Fes and Fer non-receptor protein-tyrosine kinases regulate collagen- and ADP-induced platelet aggregation. *J. Thromb. Haemost.* **1**, 1062–1070 (2003).
8. Hellwig, S. *et al.* Small-molecule inhibitors of the c-Fes protein-tyrosine kinase. *Chem. Biol.* **19**, 529–540 (2012).
9. Zou, W. *et al.* Syk, c-Src, the αvβ3 integrin, and ITAM immunoreceptors, in concert, regulate osteoclastic bone resorption. *J. Cell Biol.* **176**, 877–888 (2007).
10. Weir, M. C. *et al.* Dual inhibition of Fes and Flt3 tyrosine kinases potently inhibits Flt3-ITD+ AML cell growth. *PLoS One* **12**, e0181178 (2017).
11. Puissant, A. *et al.* SYK Is a Critical Regulator of FLT3 in Acute Myeloid Leukemia. *Cancer Cell* **25**, 226–242 (2014).
12. Roskoski, R. Properties of FDA-approved small molecule protein kinase inhibitors. *Pharmacol. Res.* **144**, 19–50 (2019).
