# Supplementary material for: Chemical genetics strategy to profile kinase target engagement reveals role of FES in neutrophil phagocytosis
Source: Nat Commun. 2020 Jun 25;11:3216. doi: 10.1038/s41467-020-17027-5 (PMC7316778; doi:10.1038/s41467-020-17027-5)
Supplement: Supplementary file 4 — Reporting Summary [file 41467_2020_17027_MOESM4_ESM.pdf]

nature research

Corresponding author(s): Prof. M. van der Stelt

Last updated by author(s): Mar 29, 2020

Reporting Summary

Nature Research wishes to improve the reproducibility of the work that we publish. This form provides structure for consistency and transparency in reporting. For further information on Nature Research policies, see Authors & Referees and the Editorial Policy Checklist .

Statistics

For all statistical analyses, confirm that the following items are present in the figure legend, table legend, main text, or Methods section.

n/a	Confirmed	
☐	☒	The exact sample size (*n*) for each experimental group/condition, given as a discrete number and unit of measurement
☐	☒	A statement on whether measurements were taken from distinct samples or whether the same sample was measured repeatedly
☐	☒	The statistical test(s) used AND whether they are one- or two-sided<br>*Only common tests should be described solely by name; describe more complex techniques in the Methods section.*
☒	☐	A description of all covariates tested
☐	☒	A description of any assumptions or corrections, such as tests of normality and adjustment for multiple comparisons
☐	☒	A full description of the statistical parameters including central tendency (e.g. means) or other basic estimates (e.g. regression coefficient) AND variation (e.g. standard deviation) or associated estimates of uncertainty (e.g. confidence intervals)
☐	☒	For null hypothesis testing, the test statistic (e.g. *F*, *t*, *r*) with confidence intervals, effect sizes, degrees of freedom and *P* value noted<br>*Give P values as exact values whenever suitable.*
☒	☐	For Bayesian analysis, information on the choice of priors and Markov chain Monte Carlo settings
☒	☐	For hierarchical and complex designs, identification of the appropriate level for tests and full reporting of outcomes
☒	☐	Estimates of effect sizes (e.g. Cohen's *d*, Pearson's *r*), indicating how they were calculated

Our web collection on statistics for biologists contains articles on many of the points above.

Software and code

Policy information about availability of computer code

Data collection	Schrödinger Suite (2017-2, Schrödinger), Prime (2017-2, Schrödinger), PyMOL Molecular Graphics System (version 1.8, Schrödinger). Also see Methods.
Data analysis	BioNavigator 6.3 (PamGene International B.V), Excel 2010 (Microsoft), Image Lab 7 (Bio-Rad), ISOQuant 1.5 (Universität Mainz), GuavaSoft InCyte 3.3 (Merck MilliPore), PLGS 3.0.3 (Waters), Prism 7/8 (GraphPad), MestReNova 9.1 (Mestrelab Research). Also see Methods.

For manuscripts utilizing custom algorithms or software that are central to the research but not yet described in published literature, software must be made available to editors/reviewers. We strongly encourage code deposition in a community repository (e.g. GitHub). See the Nature Research guidelines for submitting code & software for further information.

Data

Policy information about availability of data

All manuscripts must include a data availability statement. This statement should provide the following information, where applicable:

- Accession codes, unique identifiers, or web links for publicly available datasets
- A list of figures that have associated raw data
- A description of any restrictions on data availability

The source data underlying Figures 2-7 , Table 1, Supplementary Figures 1-2, 4, 5-9 and 12-18 and Supplementary Tables 3-4 are provided as a Source Data file. Other data are available from authors upon request.
The TempO-Seq data associated with Figure 5g are available online in the Gene Expression Omnibus (GEO) database (https://www.ncbi.nlm.nih.gov). The GEO accession number is GSE145811.
The proteomics data associated with Figure 6f-g and Figure 7c are available online in the Proteomics Identifications Database (PRIDE) (https://www.ebi.ac.uk/pride). The PRIDE dataset identifier is PXD018270.
Substrate consensus motif was generated using Enologos (http://www.benoslab.pitt.edu). The DESKGEN™ (www.deskgen.com) and CHOPCHOP v2 (http://

chopchop.cbu.uib.no) online web tools were used for CRISPR design and analyses. Uniprot databases were used for proteomics analyses (https://www.uniprot.org/proteomes).

Field-specific reporting

Please select the one below that is the best fit for your research. If you are not sure, read the appropriate sections before making your selection.

- [x] Life sciences
- [ ] Behavioural & social sciences
- [ ] Ecological, evolutionary & environmental sciences

For a reference copy of the document with all sections, see nature.com/documents/nr-reporting-summary-flat.pdf

Life sciences study design

All studies must disclose on these points even when the disclosure is negative.

Sample size	Unless stated otherwise, sample size corresponds to n = 3 measurements, except for some flow cytometry measurements (n = 5). Where applicable, biological replicates were used and in some cases measured in technical replicates. No statistical methods were used to predetermine sample size. Sample sizes were chosen based on prior knowledge in the respective experiments and their intrinsic variability as performed in previous studies (Soethoudt et al. Nat. Commun. 2017, van Esbroeck et al. Science 2017, Mock et al. Nat. Chem. Biol. 2020).
Data exclusions	No data were excluded.
Replication	Reproducibility of experiments was confirmed by the use of separately measured (biological) replicates and/or appropriate controls. In vitro biochemical and cellular experiments were performed at least in two independent experiments. Attempts at replication were successful. The exact number of replicates per data point is indicated in figure legends.
Randomization	LC-MS measurements for chemical proteomics experiments were randomized. Randomization was not applicable for other experiments. All biological and biochemical experiments were carried out with appropriate internal negative and/or positive controls as indicated.
Blinding	The investigators were not blinded, because collection or analysis of the presented data was not prone to bias. All experiments are precise (and generally quantitative) measurements of enzyme activity, protein labeling, protein expression or phoshoprotein levels and are not based on subjective assessments.

Reporting for specific materials, systems and methods

We require information from authors about some types of materials, experimental systems and methods used in many studies. Here, indicate whether each material, system or method listed is relevant to your study. If you are not sure if a list item applies to your research, read the appropriate section before selecting a response.

Materials & experimental systems

n/a	Involved in the study	
[ ]	[x]	Antibodies
[ ]	[x]	Eukaryotic cell lines
[x]	[ ]	Palaeontology
[x]	[ ]	Animals and other organisms
[x]	[ ]	Human research participants
[x]	[ ]	Clinical data

Methods

n/a	Involved in the study	
[x]	[ ]	ChIP-seq
[ ]	[x]	Flow cytometry
[x]	[ ]	MRI-based neuroimaging

Antibodies

Antibodies used	TR-FRET kinase assay:<br>- LANCE® Eu-W1024 anti-phosphotyrosine (PT66) (2 nM final concentration, PerkinElmer, AD0068)<br><br>PamChip microarray:<br>- monoclonal mouse PY20-FITC phospho-tyrosine (300 ng per array, 7.5 µg/mL final concentration, AbD Serotec/Bio-Rad, MCA2472)<br>- penta-His Alexa Fluor 488 conjugate (200 ng per array, 5 µg/mL final concentration, Qiagen, 35310)<br><br>Immunoblot:<br>- monoclonal mouse anti-FLAG M2 (1:5000, Sigma Aldrich, F3156)<br>- monoclonal anti-V5 (1:5000, Thermo Fisher, R960-25)<br>- monoclonal mouse anti-β-actin (1:1000, Abcam, ab8227)

- polyclonal rabbit anti-Lamin B1 (1:5000, Thermo Fisher, PA5-19468)
- polyclonal rabbit anti-phospho-FES Y713 (1:1000, Thermo Fisher, PA5-64504)
- monoclonal rabbit anti-FES (1:1000, Cell Signaling Technology (CST), #85704)
- polyclonal rabbit anti-phospho-SYK Y352 (1:1000, CST, #2701)
- monoclonal rabbit anti-SYK (1:1000, CST, #13198)
- polyclonal rabbit anti-phospho-HS1 Y397 (1:1000, CST, #4507)
- polyclonal rabbit anti-HS1 (1:1000, CST, #4503)
- polyclonal rabbit anti-phospho-PLCγ2 Y1217 (1:1000, CST, #3871)
- polyclonal rabbit anti-PLCγ2 (1:1000, CST, #3872).
- goat anti-mouse-HRP (1:5000, Santa Cruz, sc-2005)
- goat anti-rabbit-HRP (1:5000, Santa Cruz, sc-2030)

Flow cytometry:
- monoclonal rat CD11b-APC (1:100, Miltenyi Biotec, 130-113-231)
- rat anti-IgG2b-APC isotype control antibody (1:100, Miltenyi Biotec, 130-106-728)

Validation

The used antibodies were validated by commercial parties (PamGene) the suppliers (PerkinElmer, Sigma Aldrich, Thermo Fisher, Abcam, Cell Signaling Technology) and/or previous literature reports for the used species and applications.
- Anti-FLAG, anti-V5, anti-(phospho)-FES and anti-(phospho)-SYK were also validated using overexpressing HEK293T or U2OS lysate.
- Anti-FES antibody was additionally validated using FES-knockout HL-60 cell lysate.

Eukaryotic cell lines

Policy information about cell lines

Cell line source(s)

HEK293T, U2OS and HL-60 cells were obtained from ATCC.

Authentication

None of the cell lines were authenticated.

Mycoplasma contamination

All of the cell lines were negative for mycoplasma infection during all of our routine checks.

Commonly misidentified lines
(See ICLAC register)

None of the cell lines are present in the ICLAC register as commonly misidentified.

Flow Cytometry

Plots

Confirm that:

☒ The axis labels state the marker and fluorochrome used (e.g. CD4-FITC).

☒ The axis scales are clearly visible. Include numbers along axes only for bottom left plot of group (a 'group' is an analysis of identical markers).

☒ All plots are contour plots with outliers or pseudocolor plots.

☒ A numerical value for number of cells or percentage (with statistics) is provided.

Methodology

Sample preparation

CD11b expression measurements: Cells (1x106 per sample) were centrifuged (500 g, 3 min) and suspended in human FcR blocking solution (Miltenyi Biotec, 25x diluted in FACS buffer (1% BSA, 1% FCS, 0.1% NaN3, 2 mM EDTA in PBS)), transferred to a V-bottom 96-well plate and incubated for 10 min at 4°C. Next, monoclonal rat CD11b-APC antibody (1:100, Miltenyi Biotec, 130-113-231) or rat anti-IgG2b-APC isotype control antibody (1:100, Miltenyi Biotec, 130-106-728) was added along with 7-AAD (1 µg/mL) and samples were incubated for 30 min 4°C in the dark. Samples were washed once in PBS and fixed in 1% PFA in PBS for 15 min at 4°C in the dark, followed by two washing steps in PBS and resuspension in FACS buffer to a density of approximately 500 cells/µL.

Phagocytosis assay: Post-infection with GFP-expression E. coli, HL-60 neutrophils were resuspended and transferred to Eppendorf tubes, washed in FACS buffer (1 mL, 500 g, 3 min) and fixed in 1% PFA in PBS (15 min, 4°C, in the dark), followed by two washing steps in PBS and resuspension in FACS buffer to a density of approximately 500 cells/µL.

Instrument

Guava easyCyte HT (Merck Millipore).

Software

GuavaSoft InCyte 3.3 (Merck Millipore).

Cell population abundance

The cell population consisted only of non-differentiated and differentiated HL-60 cells and the percentage of differentiated cells is indicated in the corresponding figures.

Gating strategy

Events were gated by forward and side scatter to exclude non-singlets. The percentage of CD11b-positive cells was determined based on a treshold set for background fluorescence observed in the isotype control and non-differentiated cells. The percentage of GFP-positive neutrophils is based on background fluorescence observed in non-infected control cells. The gating strategy is exemplified in Supplementary Fig. 19.

☒ Tick this box to confirm that a figure exemplifying the gating strategy is provided in the Supplementary Information.
